# Transcriptional Profiling of Human Peripheral Blood Mononuclear Cells Identifies Diagnostic Biomarkers That Distinguish Active and Latent Tuberculosis

**DOI:** 10.3389/fimmu.2019.02948

**Published:** 2019-12-18

**Authors:** Sen Wang, Lei He, Jing Wu, Zumo Zhou, Yan Gao, Jiazhen Chen, Lingyun Shao, Ying Zhang, Wenhong Zhang

**Affiliations:** ^1^Department of Infectious Diseases, Institute of Infectious Diseases, Huashan Hospital, Fudan University, Shanghai, China; ^2^Department of Infectious Diseases, People's Hospital of Zhuji, Zhuji, China; ^3^Department of Molecular Microbiology and Immunology, Bloomberg School of Public Health, Johns Hopkins University, Baltimore, MD, United States

**Keywords:** peripheral blood mononuclear cell, biomarker, tuberculosis, latent tuberculosis infection, RNA sequence, TNFRSF10C

## Abstract

*Mycobacterium tuberculosis* (*M. tuberculosis*) infection in humans can cause active disease or latent infection. However, the factors contributing to the maintenance of latent infection vs. disease progression are poorly understood. In this study, we used a genome-wide RNA sequencing (RNA-seq) approach to identify host factors associated with *M. tuberculosis* infection status and a novel gene signature that can distinguish active disease from latent infection. By RNA-seq, we characterized transcriptional differences in purified protein derivative (PPD)-stimulated peripheral blood mononuclear cells (PBMCs) among three groups: patients with active tuberculosis (ATB), individuals with latent TB infection (LTBI), and TB-uninfected controls (CON). A total of 401 differentially expressed genes enabled grouping of individuals into three clusters. A validation study by quantitative real-time PCR (qRT-PCR) confirmed the differential expression of *TNFRSF10C, IFNG, PGM5, EBF3*, and *A2ML1* between the ATB and LTBI groups. Additional clinical validation was performed to evaluate the diagnostic performance of these five biomarkers using 130 subjects. The 3-gene signature set of *TNFRSF10C, EBF3*, and *A2ML1* enabled correct classification of 91.5% of individuals, with a high sensitivity of 86.2% and specificity of 94.9%. Diagnostic performance of the 3-gene signature set was validated using a clinical cohort of 147 subjects with suspected ATB. The sensitivity and specificity of the 3-gene set for ATB were 82.4 and 92.4%, respectively. In conclusion, we detected distinct gene expression patterns in PBMCs stimulated by PPD depending on the status of *M. tuberculosis* infection. Furthermore, we identified a 3-gene signature set that could distinguish ATB from LTBI, which may facilitate rapid diagnosis and treatment for more effective disease control.

## Introduction

Tuberculosis (TB) remains the leading cause of death as an infectious pathogen in the world. According to the World Health Organization (WHO) report, there are approximately 10.4 million new cases and 1.8 million deaths of TB each year. The interactions between *Mycobacterium tuberculosis* (*M. tuberculosis*) and the host immune responses are complex, and our understandings of the pathogenesis and protective immune responses during infection still need to be improved. Most individuals infected with *M. tuberculosis* remain asymptomatic, despite a continued immune response, a condition termed latent tuberculosis infection (LTBI). The protective immune response from host cells could presumably prevent replication of *M. tuberculosis* but fails to eradicate the pathogen (1, 2). Ten percentage of individuals with LTBI will progress to active TB during their lifetime (3). It is also possible for the pathogen to be successfully eradicated, as indicated by a loss of *M. tuberculosis*-specific effector memory T cells, which are detectable by measuring interferon-gamma (IFN-γ) production in short-term assays (4). The mechanisms underlying these differential outcomes after *M. tuberculosis* infection remain unclear, and more studies are needed to determine the immunological basis underlying active disease, latent infection, or clearance (4–6).

Until now, new diagnostic biomarkers are still urgently needed due to the lack of suitable tests to detect *M. tuberculosis* or its products directly from host samples during LTBI. The tuberculin skin test (TST) for LTBI cannot differentiate between *M. tuberculosis* infection and BCG vaccination. T cell-based IFN-γ release assays (IGRAs) assess IFN-γ production after the *in vitro* stimulation of whole blood or peripheral blood mononuclear cells (PBMCs) with *M. tuberculosis*-specific immunodominant antigens, such as 6 kDa early secretory antigenic target (ESAT6), 10 kDa culture filtrate antigen (CFP10), and TB7.7 (7, 8). A meta-analysis has shown that IGRAs have comparable sensitivity and higher specificity than the TST (9). However, current IGRAs cannot discriminate active TB (ATB) from LTBI and are therefore are particularly unsuitable for high TB endemic areas (10). New host biomarkers are needed to diagnose tuberculosis, especially to discriminate active TB from latent infection.

Cytokine responses to *M. tuberculosis*-specific antigens may differentiate active from latent TB (11–14). More recently, several genome-wide transcriptomic studies have identified differential host gene expression profiles depending on *M. tuberculosis* infection states (14–18). One study has identified nine RNA transcripts (*RIN3, LY6G6D, TEX264, C14orf2, SOCS3, KIAA2013, ASNA1, ATP5G1*, and *NOLA3*) from whole blood samples with the potential to differentiate between subjects with active and latent TB (16). Another study has shown that high-affinity IgG Fc receptor IB (*Fc*γ*RIB*) together with four other transcripts could discriminate between ATB and LTBI (18). RAS and RAB interactor 3 (*RIN3)* have also been identified as potential biomarkers of active, recurrent, cured, and latent tuberculosis (16). A recent bioinformatic analysis of public gene expression microarray data identified a set of three genes (*GBP5, DUSP3*, and *KLF2*) for diagnosis of ATB, with no clinical validation (19). However, most of these studies have used peripheral whole blood without antigen stimulation, suggesting the potential for interference with conditions other than TB. Additionally, the results may be influenced by variation in genetic background.

This study aims to better understand the immunological characteristics of different status of TB infection and to identify new transcriptional diagnostic biomarkers. Blood samples from individuals with clinically confirmed active disease and different susceptibility phenotypes were collected. Then, we performed a genome-wide transcription analysis of purified protein derivative (PPD)-stimulated PBMCs by a genome-wide RNA sequencing (RNA-seq) and identified unique transcription profiles in individuals with ATB and LTBI. A discriminatory signature gene set that differed among these groups was identified. Subsequent validation with an independent cohort finally established the discriminatory value of the gene for the discrimination between ATB and other diseases.

## Materials and Methods

### Study Design and Case Definitions

**Figure 1:**
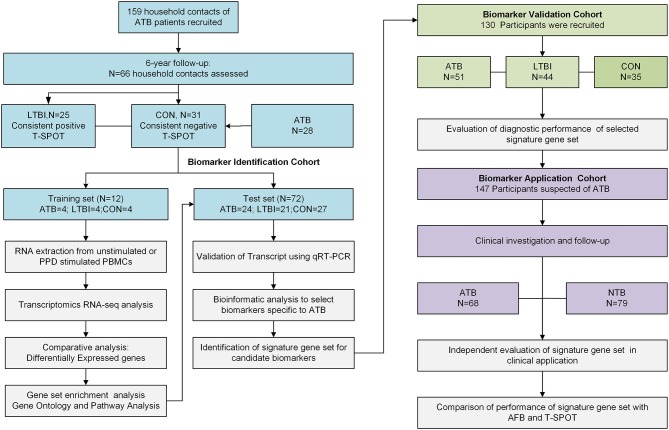
Overall study design and subjects in the Biomarker Identification, Biomarker Validation and Biomarker Application cohorts. The subjects in LTBI and CON group from Biomarker Identification Cohort were recruited from household contacts of ATB patients from a prospective study during 6-year follow up. Subjects in this cohort were assigned to either the training set or the test set randomly. Subjects in training set were selected for RNA-seq. The differentially expressed genes found by RNA-seq were tested in the test set and then validated in an independent Biomarker Validation Cohort by qRT-PCR. The identified diagnostic gene signature was then applied in the clinical-based Biomarker Application Cohort. ATB group, active TB patients; LTBI group, subjects with latent tuberculosis infection; CON group, TB-uninfected controls; NTB group, patients without ATB; qRT-PCR, quantitative real-time PCR; T-SPOT, T-SPOT®. *TB* test (Oxford Immunotec Ltd, Oxford, UK); AFB, acid-fast bacilli.

To identify and validate a reliable and effective transcriptional signature for the diagnosis of ATB, three cohorts were included in the study. The overall study design is summarized in Figure 1. General information and clinical characteristics of the recruited participants are summarized in Table 1. The study was approved by the Ethics Committee of Huashan Hospital, Fudan University and written informed consent was obtained from all participants.

**Table 1:** General information of participants in the Biomarker Identification, Biomarker Validation, and Biomarker Application Cohorts.

	**I: Biomarker Identification Cohort**			**II: Biomarker Validation Cohort**			**III: Biomarker Application Cohort**
**Characteristic**	**ATB**	**LTBI**	**CON**	**ATB**	**LTBI**	**CON**	**Patients suspected of ATB**
*N*	28	25	31	51	44	35	147
Median age (range)	41 (21–62)	43 (23–65)	39 (32–58)	45 (18–71)	43 (23–67)	41 (21–64)	44 (28–71)
Men, *n* (%)	16 (57.1%)	13 (52.0%)	16 (51.6%)	31 (60.8%)	25 (56.8%)	19 (54.3%)	83 (56.5%)
HIV infected, *n* (%)	0 (0%)	0 (0%)	0 (0%)	1 (2.0%)	0 (0%)	0 (0%)	1 (0.6%)
BCG Status							
Vaccinated	19 (67.9%)	18 (72.0%)	24 (77.4%)	36 (70.6%)	35 (79.5%)	27 (77.1%)	109 (74.1%)
Unvaccinated	9 (32.1%)	7 (28.0%)	7 (22.6%)	15 (29.4%)	9 (20.5%)	8 (22.9%)	38 (25.9%)
T-SPOT results							
Negative	3 (10.7%)	0 (0%)	31 (100%)	6 (11.8%)	0 (0%)	35 (100%)	24 (35.8%)
Positive	25 (89.3%)	25 (100%)	0 (0%)	45 (88.2%)	44 (100%)	0 (0%)	43 (64.2%)
Extrapulmonary TB	6 (21.4%)	–	–	16 (31.3%)	–	–	12 (8.2%)
Microbiologic test							
AFB positive	23 (82.1%)	0 (0%)	0 (0%)	41 (80.4%)	0 (0%)	0 (0%)	32 (21.8%)
Culture positive	20 (71.4%)	–	–	38 (74.5%)		–	46 (31.3%)

*ATB, active TB patients; LTBI, subjects with latent TB infection; CON, TB-uninfected controls*.

Subjects were assigned to diagnostic groups, i.e., patients with ATB (ATB group), subjects with latent TB infection (LTBI group), and TB-uninfected controls (CON group), independently by two experienced clinicians. The diagnosis of ATB was based on the following criteria (20): (1) clinical characteristics and symptoms including fever, cough, and productive sputum; and (2) positive acid-fast bacilli (AFB) smear and/or a positive culture for *M. tuberculosis*. Patients were all recruited less than one week before anti-TB treatment to minimize the impact of treatment on host immune responses. LTBI group were defined as subjects with positive T-SPOT®. *TB* test (Oxford Immunotec Ltd, Oxford, UK) (T-SPOT) results and with recent exposure to ATB patients but without radiological evidence of ongoing or previous ATB. In this study, all the subjects in LTBI group were recruited from household contacts of active pulmonary TB patients. The CON group was recruited from household contacts or subjects for routine physical examination and only those with no clinical or radiographic evidence of ATB and negative T-SPOT results were included.

To identify RNA-transcript signatures associated with different stages of TB infection, a discovery cohort was first used (Biomarker Identification Cohort). The LTBI and CON groups in this cohort were recruited from household contacts of patients with ATB from a 6-year follow-up study (Supplementary Figure 1) aimed at monitoring the development of active disease (21). The household contacts that had a consistent positive T-SPOT test, negative chest radiograph and no clinical symptoms or evidence of ATB within the 6-year follow up period were included in LTBI group. The household contacts with consistent negative T-SPOT results and no clinical and radiological evidence of ATB were included in the CON group (Supplementary Tables 1, 2). Subjects in this cohort were assigned to either the training set for RNA-seq or the test set for quantitative real-time PCR (qRT-PCR) validation. The RNA-seq signatures were evaluated in the test set and in an independent validation cohort (Biomarker Validation Cohort). The diagnostic performance of the validated genes signature set was finally assessed in a prospectively recruited clinical cohort (Biomarker Application Cohort) of ATB suspects and compared with that of other conventional diagnostic methods. ATB suspects are defined as patients who presented clinical symptoms (fever, night sweats, weight loss, or cough) or radiographic characteristics consistent with ATB. Further details of the study design and case definitions are provided in Supplementary Methods.

### Isolation of PBMCs and RNA Extraction

Peripheral blood (8 ml) from each participant was withdrawn from the median cubital vein of the antecubital fossa in heparinized vacutainer tubes (Becton Dickinson). PBMCs were separated within 4 h of blood withdrawal using Lympholyte Cell Separation Media (CEDARLAN, Canada). The number of viable cells was counted using Countess Automated Cell Counter (Life Technologies, USA) by trypan blue staining (14). The PBMCs from each subject were adjusted to a density of 2.5 × 10^6^ cells/ml in 1 ml of AIM-V (Life Technologies, USA) and plated in 24-well plates. The PBMCs were incubated with 10 μg/ml *M. tuberculosis* purified protein derivative (PPD, MycosResearch LLC, USA) or median alone at 37°C, 5% CO_2_ for 4 h. After 4 h, the PBMCs were harvested and suspended in TRIzol reagent (Invitrogen, CA, USA). The total RNA was immediately extracted according to the manufacturer's instructions. RNase-free DNase I (Life Technologies, USA) was used to remove genomic DNA contamination. The concentration of RNA was quantified using a NanoDrop instrument (Thermo Fisher Scientific Inc., USA). The integrity and quality of RNA were evaluated by Agilent2100 Bioanalyzer (Agilent Technologies, USA). RNA with a 2100 RIN (RNA integrity number) ≥ 7.0 and 28S/18S>0.7 was used for library preparation and RNA-seq.

### Library Preparation, Sequencing, and Data Analysis

RNA samples extracted from each group were used to generate cDNA libraries using the Illumina TruSeq RNA Sample Preparation Kit following the manufacturer's recommended procedures. Sequencing was performed on the Illumina Hiseq 2000 instrument. Detailed information on library preparation, sequencing, and data processing are provided in the Supplementary Methods.

In order to focus on differentially expressed genes among three clinical groups (ATB, LTBI, and CON group), we first calculated fold change (FC) of each gene for each individual between PPD-stimulated and unstimulated samples. Then the FC values were analyzed using the Student's *t*-test by pair-wise comparisons, including ATB vs. LTBI, ATB vs. CON, and LTBI vs. CON. The ratios of mean FC values between different groups were also calculated. Differentially expressed genes between two of the clinical groups were identified and selected for further analysis based on *P* < 0.05 by Student's *t*-test and with a ratio of > 2.0 (14, 22). The selected genes were then applied to functional analysis. The detailed bio-informatic analysis procedure is provided in the Supplementary Methods.

### qRT-PCR Analysis

The expression levels of differentially expressed genes were validated by qRT-PCR. Briefly, total RNAs were extracted from PPD-stimulated and unstimulated PBMCs using TRIzol reagent (Life Technologies, USA) according to the manufacturer's instruction. Purified RNA was reverse transcribed to cDNA using Prime-ScriptH RT reagent Kit (TaKaRa) according to the manufacturer's protocol. qRT-PCR was then performed using SYBRTM Green PCR Master Mix (TaKaRa) following standard conditions on ABI 7500 Real-time PCR System (Applied Biosystems, Inc) (14). The relative amount of expressed RNA was calculated by comparison with the expression of the housekeeping gene GAPDH using the 2^−ΔΔCt^ method (23). The qRT-PCR primers of target genes are listed in Supplementary Table 6.

### Statistical Analysis

The PPD-stimulated gene expressions were defined as fold changes by dividing the value of PPD-stimulated cells by the value of unstimulated cells. Unsupervised two-way hierarchical clustering was performed between different clinical groups for analyzing differential gene expression patterns. Mann–Whitney U tests were used to compare gene expression levels obtained by qRT-PCR between groups using SPSS 20.0 (SPSS, Chicago, IL, USA). The chi-squared test was used to compare the positive detection rate among diagnostic tests. A ROC analysis was performed to evaluate the diagnostic ability of selected genes to distinguish ATB from other diseases and the overall accuracy was assessed by area under curve (AUC) values. Combinations of markers were identified by a decision tree analysis using R 2.12.1 (R Foundation for Statistical Computing). Using this algorithm, the best tree was chosen according to the methods of a previous study (14). All statistical tests were two-sided. *P* < 0.05 was considered statistically significant.

## Results

### Differential Gene Expression Profiles in PBMCs in Response to PPD for ATB, LTBI, and CON Groups by RNA-seq

**Figure 2:**
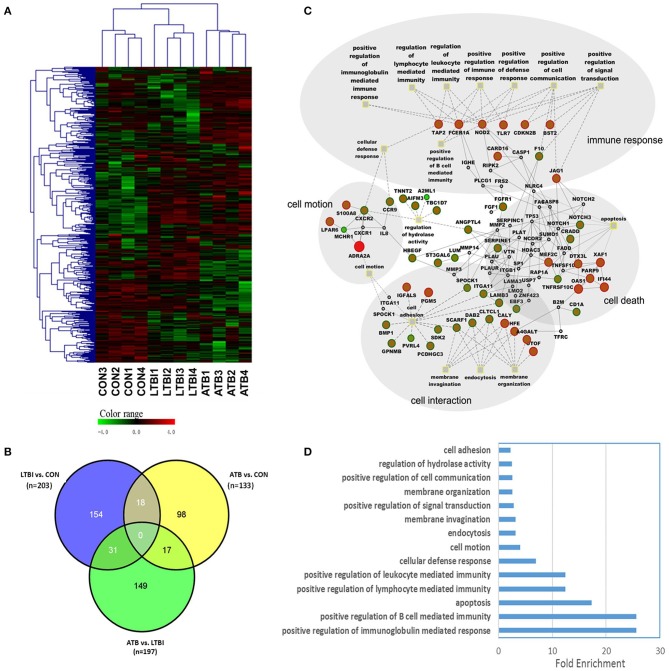
RNA sequencing (RNA-seq) analysis results and function enrichment analysis of differentially expressed genes. **(A)** Unsupervised hierarchical cluster analysis of 401 differentially expressed genes in the pair-wise comparisons. There are 4 samples in each group. Pseudocolors indicate differential expression (red, up-regulation; green, down-regulation; black, no change in expression). **(B)** Venn diagram of differentially expressed genes in PBMC samples following PPD stimulation with *P* < 0.05 by Students' *t*-test and ratio >2.0. **(C)** Regulatory network built from the differentially expressed genes between ATB and LTBI group. Circle nodes represents genes, while Gray filled rectangle nodes with yellow border color indicate biological processes. For genes, borders of the nodes represent the type of the gene (up regulated in red, down regulated in green), centers of the nodes indicate the gene expression changes, color intensity is proportional to the level of regulation. Genes that not quantified are shown in gray. Protein-protein interactions are depicted as gray solid line, dashed lines show the linkage of gene to related biological processes. Big gray circles indicate main module of biological processes. **(D)** Gene Ontology (GO) enrichment analysis for differentially regulated genes between ATB and LTBI group. Only the top false discovery rate (FDR) ranked 10 enrichment of GO terms from “biological process” category were listed.

Genome-wide transcriptional profiles of PBMCs from individuals with different stages of TB infection, including ATB, LTBI, and CON (training set in the Biomarker Identification Cohort), were determined by RNA-seq. The ratios of fold changes were determined for three pair-wise comparisons (ATB vs. LTBI, ATB vs. CON, and LTBI vs. CON). Differentially expressed genes in the three pair-wise comparisons were largely dominated by genes encoding cytokines, chemokines, and receptors. Among them, 136 differentially expressed genes had a ratio of >8 and 397 genes had a ratio of 2–8. A Venn diagram of the differentially expressed genes identified in the three pair-wise comparisons is shown in Figure 2B. Most genes were unique to a single pair-wise comparison, including 75.9% (154/203) in LTBI vs. CON, 75.6% (149/197) in ATB vs. LTBI, and 73.7% (98/133) in ATB vs. CON. After subtracting the number of shared genes among comparisons, 401 differentially expressed genes were identified in the three groups (Supplementary Table 3). An unsupervised hierarchical cluster analysis of the differentially expressed genes was used to successfully assign test individuals to three groups precisely corresponding to ATB, LTBI, and CON (Figure 2A). One large cluster containing data of all subjects with LTBI and all CON subjects was highly distinct from a cluster with data of subjects with ATB.

### Functional Categorization of Differentially Expressed Genes

According to the results of functional analysis, differentially expressed genes among ATB, LTBI, and CON groups were enriched for various GO categories. The detailed results of GO analyses are shown in Supplementary Table 4.

To identify the difference of major relevant host immune response between active tuberculosis and latent infection, we focused on the comparison between ATB and LTBI. A regulatory network was constructed using information from both a gene ontology (GO) analysis and protein–protein interaction analysis (Figure 2C). Differentially expressed genes were overrepresented for four modules in the biological process category, including immune response, cell motion, cell interaction, and cell death, providing insight into the developmental processes of the active disease vs. latent infection. The 10 most highly enriched biological processes are shown in Figure 2D. Most genes involved in immune response systems were upregulated in ATB, indicating the activation of host defense against *M. tuberculosis*. However, genes responsible for cell adhesion, membrane invasion, and endocytosis were upregulated in LTBI unlike in ATB, suggesting a possible pathway for *M. tuberculosis* survival in host cells during the latent stage. Functional categorization of differentially expressed genes between LTBI and CON group revealed that the major biological processes were immune response, regulation of apoptosis and cell death. Most genes were upregulated in LTBI group, indicating a high degree of activation of the immune response and cell death. The visualization of the regulatory network was shown in Supplementary Figure 2.

### Identification of Differentially Expressed Genes in Response to PPD Stimulation by RNA-seq

We used qRT-PCR to verify the expression levels of 37 differentially expressed genes identified by RNA-seq using a test set including 24 subjects in the ATB group, 21 in the LTBI group, and 27 in the CON group (Supplementary Table 5). The 37 genes were identified by two rounds of selection: 88 most highly differentially expressed genes in pair-wise comparisons with *P* < 0.05 by Student's *t*-test and ratio > 4 were firstly selected for pre-testing by qRT-PCR in 4 subjects with ATB, 4 with LTBI and 4 CON by qRT-PCR. Among them, 37 genes showed *P* < 0.1 in the pre-testing and were selected for further validation. For the 37 selected genes, differential expression was confirmed in 26 genes by qRT-PCR, indicating the same regulatory patterns as those identified by RNA-seq (Table 2).

**Table 2:** Significantly regulated genes in PPD-stimulated PBMCs in pair-wise comparisons validated in test set from Biomarker Identification Cohort by qRT-PCR.

**Gene symbol**	**Mean fold change**		**Ratio**	**Mann-Whitney *U***
**ATB/LTBI**	**ATB**	**LTBI**	**ATB/LTBI**	***P*-value**
IFNG	6.63	2.07	3.20	0.0341
PGM5	3.71	1.03	3.60	0.0213
EBF3	3.10	0.71	4.37	0.0151
TNFRSF10C	0.15	1.49	0.10	<0.0001
A2ML1	1.82	0.52	3.50	0.0434
**ATB/CON**	**ATB**	**CON**	**ATB/CON**	***P*****-value**
IFNG	2.07	0.71	2.91	0.0115
CXCL10	4.43	0.55	7.99	0.0185
TNFRSF10C	0.15	0.47	0.33	0.0420
ENPP3	2.54	0.53	4.75	0.0321
MYBPH	4.97	0.78	6.39	0.0124
IL26	0.73	0.22	3.38	0.0403
GPR146	0.26	1.25	0.21	0.0321
VCAN	0.32	0.87	0.37	0.0476
GPRC5A	4.61	1.05	4.39	0.0436
GPR64	0.81	2.45	0.33	0.0414
A2ML1	1.82	0.58	3.13	0.0488
EBF3	3.1	0.64	4.84	0.0231
**LTBI/CON**	**LTBI**	**CON**	**LTBI/CON**	***P*****-value**
CD1A	3.21	0.85	3.78	0.0325
HBEGF	10.93	1.74	6.30	0.0021
ZBED2	3.80	1.15	3.32	0.0263
HCAR2	5.44	1.39	3.92	0.0302
PDSS1	6.73	2.35	2.86	0.0412
KCNJ10	0.21	0.72	0.30	0.0485
IFNG	6.63	0.71	9.32	0.0051
CXCL10	4.36	0.55	7.87	0.0134
TNFRSF10C	1.49	0.47	3.20	0.0355

*Fold change: the fold change was calculated by dividing gene expression level of PPD-stimulated sample by gene expression level of unstimulated sample by qRT-PCR*.

*Ratio: the ratio was calculated by dividing mean fold change of one group by mean fold change of another group*.

Among these genes, *TNFRSF10C, IFNG, PGM5, EBF3*, and *A2ML1* exhibited statistically significant differences in the comparison of ATB vs. LTBI in PPD-stimulated PBMCs. *IFNG, EBF3, A2ML1*, and *TNFRSF10C* were also differentially expressed between ATB and CON group. *IFNG, CXCL10*, and *TNFRSF10C* were differentially expressed between ATB and CON or LTBI and CON groups. Six genes (*CD1A, HBEGF, ZBED2, HCAR2, PDSS1*, and *KCNJ10*) were differentially expressed between LTBI and CON groups. *P*-values of these in pair-wise comparisons were shown in Table 2.

### Validation of the Signature Gene Set for Discriminating ATB From LTBI in Independent Cohorts

Since the current commercially available IGRAs cannot distinguish ATB from latent infection, we focused on evaluating biomarkers that can discriminate between ATB and LTBI. For further evaluation of the differentially expressed genes between ATB and LTBI (*TNFRSF10C, IFNG, PGM5, EBF3*, and *A2ML1*), we expanded the sample size and recruited another independent set of 130 subjects, including 51 in the ATB group, 44 in the LTBI group, and 35 in the CON group (Biomarker Validation Cohort). The expression levels of the selected genes were examined by the same procedure described above.

**Figure 3:**
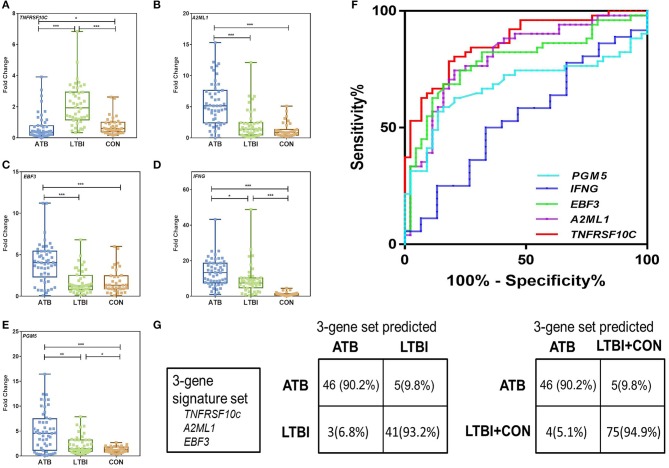
Validation of differentially expressed genes between ATB and LTBI group by qRT-PCR in the Biomarker Validation Cohort. **(A–E)** Representative scatter plots of five discriminatively expressed genes (*TNFRSF10C, IFNG, PGM5, EBF3, and A2ML1*) between ATB and LTBI were shown detected by qRT-PCR. Error bars in the scatter-dot plots indicate the medians and IQRs fold change of each group following PPD stimulation. Kruskal–Wallis tests with Dunn's post tests were used to compare the differences among three groups. *Significant difference: 0.01< *P* < 0.05; **Significant difference: 0.001< *P* < 0.01; ***Significant difference: *P* < 0.0001. **(F)** The receiver operating characteristics (ROC) curves for the five genes in discriminating between ATB and LTBI group. The ROC curves were constructed using data from subjects in ATB group as patients and subjects in LTBI group as controls. **(G)** The diagnostic performance of the 3-gene signature set in discriminating between ATB and LTBI, and between ATB and LTBI+CON in the Biomarker Validation Cohort.

Not surprisingly, the PPD-stimulated expression levels of *IFNG, PGM5, EBF3, and A2ML1* were significantly higher in the ATB group than in the LTBI and CON groups (Figures 3B–E). The results also indicated that *TNFRSF10C* was significantly downregulated after PPD stimulation in ATB group. The expression level of *TNFRSF10C* was significantly lower in the ATB group than in the LTBI and CON groups (*P* < 0.0001 and *p* = 0.021, respectively) (Figure 3A). We divided the subjects in Biomarker Validation Cohort into two subgroups based on the BCG vaccination status. There were no significant differences observed between BCG vaccinated and unvaccinated individuals for all the five genes (Supplementary Figure 3). Then we used ROC methodology to evaluate the discriminatory ability of the five genes between the ATB and LTBI groups (Supplementary Table 7). According to the optimal cutoff determined by the ROC analysis, *TNFRSF10C* had the highest AUC (0.8725, 95% CI: 0.8017–0.9434) with a sensitivity of 78.4% (40/51) and a specificity of 84.1% (37/44) for discriminating between ATB and LTBI (Figure 3F). *A2ML1* and *EBF3* also had potential diagnostic value with AUC > 0.75 (Supplementary Table 7 and Figure 3F).

We subjected these genes to a decision tree analysis to identify the best biomarker combination using R with 15-fold cross-validation. When combining the data for the LTBI and CON groups in a control group, the combination of *TNFRSF10C, A2ML1*, and *EBF3* (3-gene signature set) provided the best predictive ability, with as many as 91.5% (119/130) of individuals being correctly classified. The sensitivity and specificity of the 3-gene signature set for ATB detection were 86.2% (44/51) and 94.9% (75/79), respectively (Figure 3G).

### Clinical Application of the Optimal Signature Gene Set in Individuals With Suspected ATB

We next conducted a prospective study of 147 patients with suspected ATB (Biomarker Application Cohort). The ATB suspects enrolled were all symptomatic patients who presented with clinical or radiographic characteristics consistent with ATB before any treatment. At enrollment, all 147 patients were tested by an AFB smear, T-SPOT test, and the 3-gene signature set.

Among 147 patients, 68 met the ATB-indicative criteria (see Materials and Methods for details) and were finally diagnosed with ATB. We evaluated the diagnostic performance of the 3-gene set for classifying participants according to ATB or other diseases (including latent infection and TB-uninfected individuals). A ROC analysis was also performed for the three individual genes (Supplementary Table 8). The sensitivity, specificity and diagnostic accuracy of the 3-gene set, T-SPOT, and AFB were calculated (Table 3). The 3-gene set showed high diagnostic accuracy with a sensitivity and specificity of 82.4% (56/68) and 92.4% (73/79), respectively. The sensitivity of the 3-gene set was significantly higher than that of AFB (*P* < 0.0001). Moreover, in patients with ATB with negative AFB results (*n* = 38), the 3-gene set also showed a high sensitivity of 81.6% (31/38). The diagnostic accuracy of the 3-gene signature set showed no significant difference between BCG vaccinated and unvaccinated individuals (Supplementary Table 9). The T-SPOT test showed a high sensitivity for ATB detection (59/68, 86.8%), but a relatively low specificity (57/79, 72.2%). The specificity of the 3-gene set test was significantly higher than that of the T-SPOT test (*P* < 0.001). If the 3-gene set test was combined with AFB in a serial manner such that a positive result was defined when either of the test results was positive and a negative result was assigned when both test results were negative, the sensitivity and specificity were 89.7% (61/68) and 91.1% (72/79), respectively. The sensitivity was higher than that of the 3-gene set alone, but the difference was not significant.

**Table 3:** Diagnostic performances of the 3-gene signature set, AFB and T-SPOT in the Biomarker Application Cohort.

**Tests**	**Sensitivity%, (n/N) 95% CI**	**Specificity%, (n/N) 95% CI**	**PPV%, (n/N) 95% CI**	**NPV%, (n/N) 95% CI**	**Accuracy, (n/N) 95% CI**
AFB	44.1 (30/68)	97.5 (77/79)	93.8 (30/32)	67.0 (77/115)	73.5 (108/147)
	32.9–55.9	90.7–99.8	78.8–99.3	57.9–74.9	65.8–79.9
T-SPOT	86.8 (59/68)	72.2 (57/79)	72.8 (59/81)	86.3 (57/66)	78.9 (116/147)
	76.5–93.1	61.4–80.9	62.2–81.4	75.9–92.9	71.6–84.8
3-gene set	82.4 (56/68)	92.4 (73/79)	90.3 (56/62)	85.9 (73/85)	87.8 (129/147)
	71.5–89.8	84.1–96.8	80.1–95.8	76.8–91.9	81.4–92.2
3-gene set+AFB	89.7 (61/68)	91.1 (72/79)	89.7 (61/68)	91.1 (72/79)	90.5 (133/147)
	80.0–95.2	82.6–95.9	79.9–95.2	82.6–95.9	84.5–94.4

*PPV, positive predictive value; NPV, negative predictive value*.

*3-gene set: 3-gene signature set including TNFRSF10C, A2ML1, and EBF3*.

*3-gene set test was combined with AFB in a serial manner such that a positive result was obtained when either of the test results was positive and a negative result was assigned when both test results were negative*.

## Discussion

We identified a TB-specific transcriptional signature in PPD-stimulated PBMCs with promising diagnostic value for distinguishing ATB from other diseases with similar clinical features. This 3-gene signature set was finally evaluated using a prospective clinical cohort including patients with suspected ATB.

After infection with *M. tuberculosis*, the host could develop different outcomes including active disease, latent infection, or clearance, which depends on both bacterial and host immune factors. However, TST and commercial IGRA tests cannot distinguish latent infection from active disease (24). Previously identified biomarkers of active and latent infection vary considerably among studies, limiting their utility for clinical diagnosis (25, 26). This variation may be explained by the complexity of the infection and disease continuum as well as variation in immune responses during the infection process. Furthermore, previous studies have used un-stimulated naïve PBMCs or peripheral whole blood for gene expression profiling (17, 18). Accordingly, conditions other than *M. tuberculosis* infection could affect the TB-associated transcription profiles and candidate genes.

Several transcriptional signatures have been proposed as diagnostic biomarkers for ATB by other studies (19, 25, 27, 28). Host gene signatures for predicting progression to ATB and monitoring treatment response have also been demonstrated (29–31). However, when comparing these identified gene signatures, the results of our study showed little overlap, especially in differentially expressed genes between ATB and LTBI. Different settings (e.g., areas of different TB prevalence rates, differences in the genetic background of patients) could introduce significant variability of results among studies. Inclusion criteria for research objects (especially for LTBI and controls), and the detection method used (whole blood or antigen stimulation) are also possible explanations for this difference. Validation of our new test in a large-scale cohort including subjects from multiple sites and with different status of TB infection is still needed. In the present study, the PPD-stimulated *IFNG* expression was significantly higher in ATB group. The result is not consistent with our previous study investigating PPD-stimulated cytokine response, which showed that PPD-stimulated IFN-γ release was significantly higher in LTBI group than that in ATB group (32). The main reasons for the contrast results between studies included the differences in detection method (mRNA vs. cytokine) and the impact of incubation time on host immune response (4 h vs.16–20 h). The heterogeneity of ATB patients and subjects with LTBI could also affect the *IFNG* expression, which needs to be further explored.

In this study, we used PPD-stimulated PBMCs to minimize unrelated background noise and to simultaneously maximize *M. tuberculosis-specific* host immune responses. Compared with other studies using unstimulated whole blood or ESAT-6/CFP-10 stimulation, PPD stimulation could provoke more diverse host immune responses, which could improve the discriminatory capacity of the transcriptional gene profiles. More importantly, several other studies and our own have demonstrated that PPD-stimulated gene expressions or cytokine profiles have diagnostic potential for distinguishing between ATB and LTBI (13, 32–35). Therefore, evaluating the PPD-induced transcriptional profiles in different populations could provide more comprehensive immune factors for specific ATB and LTBI detection. It is worth noting that PBMC isolation and PPD stimulation procedures of our 3-gene signature test make it not suitable for a point-of-care test, which could not totally meet the needs for large-scale screening or application in resource-limited areas. Therefore, the detection method needs to be further simplified and optimized for adaption to a feasible and low-cost test.

Additionally, PPD shares several antigens with BCG, and a cross-reaction may occur between PPD based tests and BCG vaccination. However, our results indicated that the expression levels of the identified genes showed no significant difference between BCG vaccinated and unvaccinated individuals. BCG vaccination status did not change the diagnostic accuracy of the 3-gene signature set for discriminating ATB from other diseases. Such potential influence could also be avoided by combining with current IGRAs such as T-SPOT assay in a serial way (32). Further study with larger sample size is necessary to evaluate the impact of BCG vaccination on the diagnostic performance of the new test.

Another unique feature of the study is that we recruited household contacts of patients with smear-positive ATB, which is an effective strategy for studying the differences in immune mechanisms in response to TB infection. After 6 years of follow-up, the overall incidence of active disease in our household contact cohort was 11.0 per 1000 person-years; the vast majority of household contacts did not develop active disease and remained asymptomatic. The immune system of these subjects may control or eradicate the bacteria, despite frequent and long-term exposure to patients with active pulmonary TB. Therefore, gene expression analyses of these subjects may help to identify profiles that are correlated with disease resistance and susceptibility. In this study, the T-SPOT test was used to screen household contacts with latent infection. Owing to a high BCG-vaccination rate in this area (36), T-SPOT tests were more specific than the commonly used TST (37, 38). Moreover, with known exposure to patients with smear-positive pulmonary TB, T-SPOT-positive results for household contacts were considered to indicate a recently acquired infection, while IGRA-positive results without established TB exposure suggest remote infection (24, 39). In the Biomarker Identification Cohort, subjects with LTBI were recruited from household contacts of ATB patients with consistent positive T-SPOT results during 6-year follow-up, which may be considered as long-standing LTBI rather than recent LTBI. Since the heterogeneity of LTBI could result in different transcriptional profiles between remote and recent infection (5, 25), our 3-gene signature set might be more appropriate for diagnosing long-standing LTBI rather than recent infection. Further study will recruit subjects with different status of LTBI to validate our new biomarkers.

*TNFRSF10C* was the most highly differentially expressed gene among the three groups in this study. *TNFRSF10C* levels were significantly lower in the ATB group and higher in the LTBI group than in the control group, suggesting that its expression is regulated by host cells in a stage-specific manner. The TNFRSF10C protein, also known as TNF-Related Apoptosis-Inducing Ligand Receptor 3 (TRAIL-R3), is a member of the TNF-receptor superfamily. TRAIL-R3 contains an extracellular TRAIL-binding domain and a transmembrane domain, but no death domain in the cell. Therefore, this receptor is considered to function as an antagonistic receptor, which could protect cells from TRAIL-induced apoptosis (40, 41). The overexpression of TRAIL-R3 could avoid the TRAIL-induced cell death, which was proved in several tumor cell lines and primary tumors (42, 43). In our study, *TNFRSF10C* expression was decreased in patients with ATB, potentially eliminating its anti-apoptotic effect; this is probably a necessary step for protection against bacterial survival and growth (44–46). An alternative explanation for the elevated *TNFRSF10C* expression in the LTBI group is direct inhibition by *M. tuberculosis* infection. The significant downregulation of *TNFRSF10C* could be a survival mechanism for *M. tuberculosis* during latent infection. Future studies are needed to address these possibilities and to analyze the molecular mechanisms underlying the regulation of *TNFRSF10C*.

The present study had some limitations. First, the sample size for validation was relatively small, and future studies should evaluate the biomarkers with larger samples, focusing on patients with ATB who have immunodeficient conditions, such as subjects with HIV infection, or patients receiving immunosuppressive therapy for autoimmune diseases. Second, more symptomatic individuals suspected of ATB should be recruited for further evaluation of the new test in clinical application. Third, a small number of ATB patients in our study received anti-TB treatment for <1 week before recruitment. It has been demonstrated that the host transcriptional profiles can change rapidly over time during treatment (25, 47). Although the impact appeared to be limited within 1 week of treatment (24), further validation of the 3-gene signature is still necessary. Besides, the candidate biomarkers should be evaluated with an emphasis on the predictive value for progression to active disease in a large cohort of subjects at high risk for TB infection. Finally, functional studies are needed to determine the roles of the identified biomarkers in the pathogenesis of TB infection.

In summary, we comprehensively analyzed transcription profiles associated with different TB infectious statuses and confirmed that a 3-gene signature set (*TNFRSF10C, A2ML1*, and *EBF3*) could be used as a reliable diagnostic biomarker for ATB. These findings have important implications for the development of novel diagnostic tests to discriminate ATB from other diseases. The results could also help us get a better understanding of immune mechanisms underlying latent infection or progression to active disease after *M. tuberculosis* infection and the key immunological factors of this transformation.

## Data Availability Statement

The datasets generated for this study can be found in the NCBI Sequence Read Archive PRJNA566142: https://www.ncbi.nlm.nih.gov/bioproject/PRJNA566142.

## Ethics Statement

The studies involving human participants were reviewed and approved by Ethics Committee of Huashan Hospital, Fudan University. The patients/participants provided their written informed consent to participate in this study.

## Author Contributions

SW, LS, YZ, and WZ contributed conception and design of the study. LH, JC, and ZZ organized the database. SW, JW, and LS performed the statistical analysis. SW, LH, JW, YG, and LS collected samples and performed lab work. SW and LH wrote the draft of the manuscript. YZ and WZ revised the manuscript. All authors contributed to manuscript revision, read and approved the submitted version.

### Conflict of Interest

The authors declare that the research was conducted in the absence of any commercial or financial relationships that could be construed as a potential conflict of interest.
