# Supplementary material for: Transcriptional Profiling of Human Peripheral Blood Mononuclear Cells Identifies Diagnostic Biomarkers That Distinguish Active and Latent Tuberculosis
Source: Front Immunol. 2019 Dec 18;10:2948. doi: 10.3389/fimmu.2019.02948 (PMC6930242; doi:10.3389/fimmu.2019.02948)
Supplement: Supplementary file 1 [file Data_Sheet_1.docx]

Supplementary Material

# Supplementary Methods

## Study Sites

All the subjects in ATB, LTBI and CON group in this study were recruited from there study sites in China. The three sites included: **Site A**: Chongqing Pulmonary Hospital, Chongqing, China (Southwest China); **Site B**: Zhuji Renmin Hospital, Zhejiang, China (East China); **Site C**: Huashan Hospital, Shanghai, China (East China)

## Biomarker Identification Cohort (n=84)

- 1. **ATB group (n=28):** All the ATB patients were recruited from Chongqing Pulmonary Hospital, Chongqing, China (Study Site A) from February 2013 to April 2013. 21 patients were tested prior to the initiation of therapy (including 4 patients for RNA-seq); 4 patients received therapy for ≤ 3 days; 3 patients received therapy for 4-7 days.
  2. **LTBI group (n=25):** The household contacts of ATB patients in Study Site A were recruited and offered T-SPOT and TST test and followed 6 years (from December 2006 to January 2013) for TB development. According to the results of the T-SPOT test, the subjects who had a consistent positive T-SPOT test, negative chest radiograph and no clinical symptoms or evidence of active TB within the 6-year follow up period were included in this group.
  3. **CON group (n=31):** The household contacts with consistent negative T-SPOT results and no clinical and radiographic evidence of ATB were included in this group.

## Biomarker Validation Cohort (n=130)

- 1. **ATB group (n=51)**: 21 of the ATB patients were recruited from Chongqing Pulmonary Hospital (Study Site A) from October 2013 to December 2013, and 30 of the ATB patients were recruited from Zhuji Renmin Hospital (Study Site B) from February 2014 to June 2014. 37 patients were tested before the initiation of therapy; 8 patients received therapy for ≤ 3 days; 6 patients received therapy for 4-7 days.
  2. **LTBI group (n=44)**: The subjects were all household contacts of ATB patients in Study Site A and B from October 2013 to July 2014. T-SPOT tests and routine radiographic evaluations were performed on all household contacts. Subjects who displayed a positive QFT test, negative chest radiography and no clinical symptoms or evidence of ATB were included.
  3. **CON group (n=35)**: 15 subjects in this group were recruited from subjects who came to Huashan hospital (Study Site C) from August 2014 to October 2014 for an unrelated health examination. Another 20 subjects were recruited from household contacts in Study Site B from February 2014 to June 2014. The household contacts all displayed negative T-SPOT results, negative chest radiography and no clinical symptoms or evidence of ATB.

## Biomarker Application Cohort (n=147)

- 1. **ATB group (n=68)**: ATB suspects were recruited in Huashan Hospital (Study Site C) who presented clinical symptoms (fever, night sweats, weight loss, or cough) or radiographic characteristics consistent with ATB from March 2015 to August 2015. After a follow-up of at least 3 months, ATB was finally diagnosed using the same criteria as described above. All 68 ATB patients were tested before the initiation of ATB therapy
  2. **NTB group (n=79)**: The ATB suspects who did not meet the ATB criteria and had some other explanations of symptoms were identified as subjects without ATB disease (NTB).

## Conventional Laboratory Test for TB

Two spontaneous or induced sputum samples were examined by standard microscopy for acid-fast bacilli (AFB) and cultured for mycobacteria using the BD MGIT liquid culture technique.

## T-SPOT^®^.*TB* test (T-SPOT)

All recruited candidates were screened by T-SPOT**^®^**.*TB* kit (Oxford Immunotec Ltd, Oxford, UK). The T-SPOT test was performed according to the manufacturer’s instructions. Briefly, a precoated IFN-γ ELISPOT plate was seeded with 2.5 × 10^5^ peripheral blood mononuclear cells (PBMCs) per well and incubated with medium (Nil), peptide antigens derived from ESAT-6 (Panel A), CFP-10 (Panel B), or phytohemagglutinin (PHA, as a positive control) in a 5% CO_2_ atmosphere at 37 °C for 16–24 h. The plate wells were then washed and incubated with a conjugate against the antibody used and an enzyme substrate. Spot-forming cells (SFCs) in T-SPOT Panel A (T-SPOT A) and T-SPOT Panel B (T-SPOT B), representing antigen-specific T-cells secreting IFN-γ, were counted with an automated ELISPOT reader (AID-GmbH, Germany). The test result was considered positive if Panel A – Nil and/or Panel B – Nil ≥8 spots, and negative if both Panel A – Nil and Panel B – Nil ≤4 spots. Results were considered borderline if the Panel – Nil spot count was 5, 6, or 7 spots and was re-tested by collecting another patient specimen. The results were double-checked by other laboratory workers and, if necessary, corrected by manual counting. The laboratory technicians were blinded to the subject identifiers.

## Library preparation and RNA-seq

Total RNA was extracted from PPD-stimulated and unstimulated PBMCs using TRIzol reagent (Life Technologies, USA) according to the protocols recommended by the manufacturer. 5 µg total RNA was used to prepare the sequencing library by Illumina TrueSeq RNA Sample Preparation Kit as the manufacturer’s instruction. In brief, mRNA was purified from total RNA using oligo-dT attached magnetic beads. Following purification, the mRNA is fragmented into small pieces using divalent cations under elevated temperature. The cleaved RNA fragments are copied into first strand cDNA using reverse transcriptase and random primers. Second strand cDNA synthesis was subsequently performed using DNA Polymerase I and RNase H. After the end repair process and adenylation of 3ʹ end tailing, adapters were ligated to the cDNA. The products are purified using the AMPure XP system (Beckman Coulter, USA) and enriched with PCR to create the final cDNA library. The quality of the cDNA libraries was accessed using Agilent Technologies 2100 Bioanalyzer.

The libraries were sequenced on the Illumina Hiseq 2000 instrument according to the manufacturer’s instructions. Sequencing was carried out using a 100-nt paired-end configuration, and 63 million raw reads for each sample were obtained on average (Supplementary Table 3). Raw reads from the image data output from the sequencing machine were generated by Base Calling and saved in FASTQ format. Clean reads were generated by removing reads with adaptors, reads where the number of unknown bases was more than 10% and low-quality reads which have more than 50% QA≤15 bases. For each sample, the clean and trimmed mRNA sequencing reads were aligned to the UCSC human reference genome (hg19) using Tophat (version 2.0.6) [[1](#_ENREF_1), [2](#_ENREF_2)] with default parameters. TopHat allows a maximum of two mismatches when mapping the reads to the reference genome. The aligned reads were further subjected to Cufflinks (version 2.2.1) [[3](#_ENREF_3)] software for assembling transcripts, and the expression level for each transcript was calculated using the fragments per kilobase of exon per million mapped reads (FPKM) method. The library preparation and RNA-Seq were performed by Shanghai Biotechnology Co. Ltd.

## Differential Expression Analysis

The differentially expressed genes among the three different clinical groups (ATB, LTBI, and CON) were identified in the following steps: 1. the fold change (FC) of each gene for each individual was calculated by dividing the FPKM value of PPD–stimulated samples by the FPKM value of unstimulated control samples (FC= FPKM _PPD-stimulated_/ FPKM _unstimulated_), and defined as PPD-stimulated gene expression; 2. The mean FC value of the 4 samples in each clinical group was calculated and then compared to other groups by calculating the ratios (Ratio= mean FC of Group I/ mean FC of Group II; 3. Student's *t*-test was used to compare mean PPD-stimulated gene expression (FCs) in pair-wise comparisons included ATB vs. LTBI, ATB vs. CON, and LTBI vs. CON; 4. Differentially expressed genes were considered significant at a *P*-value < 0.05 by Student’s *t*-test and a ratio > 2.0.

## Functional annotation of differentially expressed genes

Official gene names were used to perform the gene ontology enrichment analysis. The gene ontology analysis was performed with the functional annotation tool at the DAVID bioinformatics server [[4](#_ENREF_4), [5](#_ENREF_5)], the most relevant term of biological process, cell component and molecular function were associated with the query genes. Default setting with EASE score=0.1 were used. The enriched biological processes were taken for the model construction. The information of Protein-protein interaction were obtained via the STRING database (version 9.1) [[6](#_ENREF_6)], default setting with combined score> 0.4, but no text mining was applied. For our dataset, few direct interactions exist among the query genes. The build-in one step expand algorithm is used to discover the potential indirect interactions. 3-steps extension of the network were performed. To expose the potential relationship with the differential expressed genes with disease development, network based regulation model was constructed with both information gained in gene ontology analysis and Protein-protein interaction analysis with the cytoscape [[7](#_ENREF_7)].

# Supplementary Tables

**Supplementary Table 1.** T-SPOT results of the 12 subjects (ATB=4, LTBI=4, CON=4) in the training set for RNA-seq.

| **Detection Time** | **T-SPOT** | **LTBI** | | | |  | **CON** | | | |  | **ATB** | | | |
| --- | --- | --- | --- | --- | --- | --- | --- | --- | --- | --- | --- | --- | --- | --- | --- |
|  |  | **CQ56** | **CQ104** | **CQ106** | **CQ108** |  | **CQ14** | **CQ32** | **CQ36** | **CQ119** |  | **WX1** | **WX2** | **WX3** | **WX4** |
| 2006.12 | N | 2 | 0 | 1 | 0 |  | 0 | 0 | 1 | 0 |  | − | − | − | − |
|  | A | 38 | 80 | 19 | 10 |  | 0 | 1 | 0 | 0 |  | − | − | − | − |
|  | B | 4 | 41 | 107 | 44 |  | 0 | 1 | 0 | 0 |  | − | − | − | − |
|  | M | 534 | 475 | 597 | 578 |  | 358 | 341 | 532 | 472 |  | − | − | − | − |
|  | Results | Positive | Positive | Positive | Positive |  | Negative | Negative | Negative | Negative |  | − | − | − | − |
| 2007.11 | N | 0 | 2 | 1 | 0 |  | 0 | 0 | 0 | 0 |  | − | − | − | − |
|  | A | 29 | 91 | 34 | 21 |  | 0 | 1 | 0 | 0 |  | − | − | − | − |
|  | B | 11 | 28 | 87 | 54 |  | 0 | 0 | 1 | 0 |  | − | − | − | − |
|  | M | 357 | 453 | 531 | 569 |  | 334 | 376 | 563 | 379 |  | − | − | − | − |
|  | Results | Positive | Positive | Positive | Positive |  | Negative | Negative | Negative | Negative |  | − | − | − | − |
| 2009.11 | N | 0 | 1 | 1 | 0 |  | 0 | 2 | 0 | 1 |  | − | − | − | − |
|  | A | 57 | 79 | 54 | 8 |  | 0 | 0 | 0 | 0 |  | − | − | − | − |
|  | B | 9 | 32 | 135 | 37 |  | 1 | 0 | 0 | 0 |  | − | − | − | − |
|  | M | 441 | 462 | 421 | 643 |  | 394 | 298 | 453 | 462 |  | − | − | − | − |
|  | Results | Positive | Positive | Positive | Positive |  | Negative | Negative | Negative | Negative |  | − | − | − | − |
| 2012.12 | N | 0 | 0 | 2 | 1 |  | 0 | 0 | 0 | 0 |  | 1 | 0 | 2 | 0 |
|  | A | 44 | 89 | 47 | 17 |  | 0 | 2 | 0 | 0 |  | 145 | 79 | 156 | 34 |
|  | B | 10 | 53 | 121 | 49 |  | 0 | 0 | 1 | 0 |  | 31 | 33 | 241 | 274 |
|  | M | 423 | 412 | 541 | 541 |  | 427 | 431 | 467 | 592 |  | 484 | 387 | 591 | 543 |
|  | Results | Positive | Positive | Positive | Positive |  | Negative | Negative | Negative | Negative |  | Positive | Positive | Positive | Positive |

N: negative control; A: Panel A stimulated responses; B: Panel B stimulated responses; M: PHA stimulated responses.

The numbers of spot-forming cells (SFCs) were shown in the table.

**Supplementary Table 2.** T-SPOT results of household contacts during 6-year follow-up in the test set from Biomarker Identification Cohort**.**

| **Household contacts**  **n=47** | **T-SPOT results** | |  | |  | |  |
| --- | --- | --- | --- | --- | --- | --- | --- |
|  | **2006.12** | **2007.11** | | **2009.11** | | **2012.12** | |
| CQ01 | N | N | | N | | N | |
| CQ02 | N | N | | N | | N | |
| CQ04 | N | N | | N | | N | |
| CQ06 | N | N | | N | | N | |
| CQ07 | N | N | | N | | N | |
| CQ09 | N | N | | N | | N | |
| CQ10 | N | N | | N | | N | |
| CQ11 | N | N | | N | | N | |
| CQ12 | P | P | | P | | P | |
| CQ23 | P | P | | P | | P | |
| CQ29 | P | P | | P | | P | |
| CQ31 | P | P | | P | | P | |
| CQ33 | N | N | | N | | N | |
| CQ34 | N | N | | N | | N | |
| CQ35 | P | P | | P | | P | |
| CQ37 | N | N | | N | | N | |
| CQ42 | P | P | | P | | P | |
| CQ43 | P | P | | P | | P | |
| CQ46 | N | N | | N | | N | |
| CQ48 | N | N | | N | | N | |
| CQ49 | P | P | | P | | P | |
| CQ51 | P | P | | P | | P | |
| CQ54 | N | N | | N | | N | |
| CQ57 | N | N | | N | | N | |
| CQ61 | P | P | | P | | P | |
| CQ78 | N | N | | N | | N | |
| CQ79 | N | N | | N | | N | |
| CQ82 | N | N | | N | | N | |
| CQ92 | P | P | | P | | P | |
| CQ95 | P | P | | P | | P | |
| CQ96 | N | N | | N | | N | |
| CQ102 | N | N | | N | | N | |
| CQ105 | P | P | | P | | P | |
| CQ107 | P | P | | P | | P | |
| CQ109 | P | P | | P | | P | |
| CQ112 | N | N | | N | | N | |
| CQ114 | N | N | | N | | N | |
| CQ118 | P | P | | P | | P | |
| CQ123 | P | P | | P | | P | |
| CQ129 | N | N | | N | | N | |
| CQ137 | N | N | | N | | N | |
| CQ141 | P | P | | P | | P | |
| CQ143 | N | N | | N | | N | |
| CQ147 | P | P | | P | | P | |
| CQ148 | N | N | | N | | N | |
| CQ151 | P | P | | P | | P | |
| CQ152 | P | P | | P | | P | |

N: negative; P: positive

**Supplementary Table 3.** Read lengths and qualities of RNA-seq.

| **Sample Name** | **Seq. type** | **Orientation** | **Raw reads (M)** | **Raw bases(G)** | **Q20 Value(%)** | **Clean reads (M)** |
| --- | --- | --- | --- | --- | --- | --- |
| CQ104- | RNA | F/R | 83 | 8.3 | 95.3 | 70.9 |
| CQ104-PPD | RNA | F/R | 104 | 10.4 | 95.2 | 87.8 |
| CQ106- | RNA | F/R | 103.6 | 10.4 | 94.8 | 86.4 |
| CQ106-PPD | RNA | F/R | 79.3 | 7.9 | 95.4 | 67.3 |
| CQ108- | RNA | F/R | 61.2 | 6.1 | 94.9 | 51.2 |
| CQ108-PPD | RNA | F/R | 49.1 | 4.9 | 95.1 | 41.2 |
| CQ119- | RNA | F/R | 51.2 | 5.1 | 95.8 | 44.2 |
| CQ119-PPD | RNA | F/R | 127.5 | 12.8 | 95.4 | 108.2 |
| CQ14- | RNA | F/R | 53.3 | 5.3 | 95.9 | 45.8 |
| CQ14-PPD | RNA | F/R | 35.9 | 3.6 | 95.5 | 30.1 |
| CQ32- | RNA | F/R | 49.3 | 4.9 | 95.7 | 42.2 |
| CQ32-PPD | RNA | F/R | 43.4 | 4.3 | 95.6 | 36.5 |
| CQ36- | RNA | F/R | 77.2 | 7.7 | 95.7 | 65.8 |
| CQ36-PPD | RNA | F/R | 60.2 | 6 | 95.7 | 51.3 |
| CQ56- | RNA | F/R | 81.3 | 8.1 | 94.8 | 67.7 |
| CQ56-PPD | RNA | F/R | 40.9 | 4.1 | 94.8 | 34.2 |
| WX1- | RNA | F/R | 47.4 | 4.7 | 94.7 | 39.4 |
| WX1-PPD | RNA | F/R | 63.2 | 6.3 | 95.2 | 53.0 |
| WX2- | RNA | F/R | 38.5 | 3.9 | 95 | 32.4 |
| WX2-PPD | RNA | F/R | 68.4 | 6.8 | 95.1 | 57.6 |
| WX3- | RNA | F/R | 72 | 7.2 | 95.1 | 61.0 |
| WX3-PPD | RNA | F/R | 42.5 | 4.3 | 95.2 | 35.9 |
| WX4- | RNA | F/R | 35.4 | 3.5 | 95.1 | 29.7 |
| WX4-PPD | RNA | F/R | 49.4 | 4.9 | 95.4 | 41.9 |

Q20=bases of Q>=20 / all bases of sequencing

**Supplementary Table 4.** Significantly regulated genes in PPD-stimulated PBMCs in pair-wise comparisons with ratio >2 and *P*-value <0.05 by RNA-seq.

| **Gene Symbol** | **Mean fold change** | | **Ratio** | **Regulation** | ***P* value** |
| --- | --- | --- | --- | --- | --- |
| **ATB/LTBI** | **ATB** | **LTBI** | **ATB/LTBI** |  |  |
| ADRA2A | 1.46 | 0.05 | 30.77 | up | 0.0367 |
| A2ML1 | 10.20 | 0.40 | 25.32 | up | 0.0307 |
| PGM5 | 4.73 | 0.37 | 12.90 | up | 0.0495 |
| KDM4E | 1.85 | 0.15 | 12.62 | up | 0.0014 |
| OAS1 | 12.04 | 1.11 | 10.83 | up | 0.0452 |
| IFI44 | 13.05 | 1.27 | 10.31 | up | 0.0494 |
| CCSER1 | 2.93 | 0.31 | 9.55 | up | 0.0186 |
| XAF1 | 9.05 | 1.30 | 6.96 | up | 0.0261 |
| DCST1 | 1.43 | 0.21 | 6.69 | up | 0.0292 |
| CALY | 0.25 | 0.04 | 6.06 | up | 0.0112 |
| FCER1A | 1.54 | 0.26 | 6.03 | up | 0.0370 |
| OTOF | 1.91 | 0.32 | 5.86 | up | 0.0260 |
| EBF3 | 1.63 | 0.29 | 5.71 | up | 0.0191 |
| MEG3 | 2.11 | 0.37 | 5.66 | up | 0.0366 |
| SCGB1B2P | 2.08 | 0.37 | 5.59 | up | 0.0126 |
| RBMS3 | 1.82 | 0.33 | 5.58 | up | 0.0181 |
| TAP2 | 2.84 | 0.59 | 4.78 | up | 0.0194 |
| PVALB | 3.41 | 0.81 | 4.22 | up | 0.0095 |
| FGL2 | 0.55 | 0.13 | 4.13 | up | 0.0173 |
| MYOZ3 | 2.38 | 0.62 | 3.86 | up | 0.0410 |
| TLR7 | 3.57 | 0.93 | 3.84 | up | 0.0226 |
| RUFY4 | 0.81 | 0.21 | 3.80 | up | 0.0127 |
| CARD17 | 2.94 | 0.78 | 3.77 | up | 0.0459 |
| SLC6A9 | 6.89 | 1.85 | 3.73 | up | 0.0493 |
| UNC93B1 | 2.01 | 0.57 | 3.50 | up | 0.0484 |
| SLC24A3 | 0.79 | 0.24 | 3.37 | up | 0.0215 |
| PGBD5 | 0.84 | 0.26 | 3.20 | up | 0.0128 |
| PARP9 | 6.42 | 2.02 | 3.18 | up | 0.0351 |
| ODF3B | 1.41 | 0.46 | 3.03 | up | 0.0327 |
| TMEM200A | 5.77 | 1.92 | 3.00 | up | 0.0258 |
| FCRL1 | 6.09 | 2.04 | 2.98 | up | 0.0132 |
| HFE | 0.83 | 0.28 | 2.98 | up | 0.0013 |
| BST2 | 3.41 | 1.15 | 2.97 | up | 0.0495 |
| AHNAK2 | 0.82 | 0.28 | 2.94 | up | 0.0008 |
| CMTM8 | 2.12 | 0.73 | 2.91 | up | 0.0024 |
| CRYBA4 | 0.69 | 0.24 | 2.82 | up | 0.0483 |
| CR1L | 0.41 | 0.15 | 2.69 | up | 0.0316 |
| TMEM26 | 1.41 | 0.53 | 2.65 | up | 0.0262 |
| JAG1 | 4.32 | 1.64 | 2.64 | up | 0.0032 |
| FAM127C | 2.57 | 0.98 | 2.63 | up | 0.0296 |
| C7orf10 | 1.63 | 0.63 | 2.56 | up | 0.0187 |
| CABP4 | 0.93 | 0.38 | 2.43 | up | 0.0027 |
| IGFALS | 2.03 | 0.84 | 2.41 | up | 0.0415 |
| CDKN2B | 1.79 | 0.74 | 2.40 | up | 0.0151 |
| PCK2 | 1.57 | 0.66 | 2.37 | up | 0.0124 |
| TCTEX1D1 | 0.94 | 0.40 | 2.37 | up | 0.0259 |
| LINC00854 | 1.84 | 0.79 | 2.32 | up | 0.0127 |
| A4GALT | 1.55 | 0.67 | 2.32 | up | 0.0389 |
| DISC1 | 1.90 | 0.83 | 2.29 | up | 0.0155 |
| DTX3L | 5.05 | 2.22 | 2.27 | up | 0.0438 |
| HIST4H4 | 1.09 | 0.48 | 2.24 | up | 0.0240 |
| GCSAML | 2.10 | 0.95 | 2.22 | up | 0.0258 |
| LPAR6 | 0.79 | 0.36 | 2.17 | up | 0.0448 |
| NOD2 | 1.69 | 0.78 | 2.16 | up | 0.0425 |
| UBQLNL | 2.06 | 0.96 | 2.15 | up | 0.0327 |
| CPA3 | 1.29 | 0.61 | 2.12 | up | 0.0039 |
| S100A8 | 1.19 | 0.57 | 2.10 | up | 0.0487 |
| STAC3 | 0.82 | 0.39 | 2.10 | up | 0.0055 |
| PAK3 | 1.09 | 0.52 | 2.09 | up | 0.0489 |
| PRSS36 | 0.28 | 0.13 | 2.08 | up | 0.0435 |
| MEF2C | 0.73 | 0.35 | 2.07 | up | 0.0395 |
| ZMYM4-AS1 | 1.24 | 0.62 | 2.01 | up | 0.0269 |
| CARD16 | 1.78 | 0.89 | 2.00 | up | 0.0450 |
| MCHR1 | 0.03 | 0.99 | 35.90 | down | 0.0143 |
| CALHM1 | 0.03 | 0.83 | 30.30 | down | 0.0221 |
| PVRL4 | 0.06 | 1.51 | 26.92 | down | 0.0439 |
| APOC4 | 0.00 | 0.06 | 12.75 | down | 0.0325 |
| SPOCK1 | 0.24 | 2.79 | 11.68 | down | 0.0489 |
| CD1A | 0.47 | 4.39 | 9.36 | down | 0.0237 |
| TNFRSF10C | 0.11 | 0.97 | 8.93 | down | 0.0067 |
| LUM | 0.28 | 2.19 | 7.75 | down | 0.0405 |
| LRRC36 | 0.25 | 1.73 | 7.03 | down | 0.0018 |
| ITGA11 | 0.02 | 0.13 | 6.10 | down | 0.0119 |
| GSTM5 | 0.15 | 0.89 | 5.87 | down | 0.0104 |
| SDK2 | 0.33 | 1.92 | 5.75 | down | 0.0199 |
| SHROOM3 | 1.43 | 7.89 | 5.52 | down | 0.0171 |
| ZNF843 | 0.43 | 2.25 | 5.24 | down | 0.0269 |
| LDLRAD2 | 0.21 | 1.06 | 5.05 | down | 0.0187 |
| FAM227A | 0.43 | 1.91 | 4.47 | down | 0.0464 |
| NAV2-AS1 | 0.29 | 1.26 | 4.42 | down | 0.0304 |
| CD300LB | 0.08 | 0.33 | 4.20 | down | 0.0394 |
| SLC28A3 | 0.33 | 1.40 | 4.19 | down | 0.0251 |
| RFX8 | 1.44 | 6.01 | 4.18 | down | 0.0186 |
| CXCL9 | 1.31 | 5.33 | 4.07 | down | 0.0436 |
| HBEGF | 5.64 | 22.72 | 4.03 | down | 0.0471 |
| CMTM2 | 0.26 | 1.01 | 3.95 | down | 0.0334 |
| CLEC3B | 0.22 | 0.88 | 3.90 | down | 0.0158 |
| KPTN | 0.83 | 3.16 | 3.82 | down | 0.0211 |
| SLC9B1 | 0.98 | 3.63 | 3.72 | down | 0.0119 |
| DNAJB13 | 0.09 | 0.33 | 3.70 | down | 0.0482 |
| STOX2 | 0.35 | 1.27 | 3.60 | down | 0.0180 |
| SYBU | 0.38 | 1.29 | 3.41 | down | 0.0011 |
| SHANK1 | 0.47 | 1.54 | 3.27 | down | 0.0199 |
| C19orf59 | 1.32 | 4.27 | 3.24 | down | 0.0488 |
| TCL6 | 0.53 | 1.71 | 3.24 | down | 0.0103 |
| FBXO43 | 0.56 | 1.73 | 3.07 | down | 0.0366 |
| ANGPTL4 | 0.15 | 0.44 | 3.03 | down | 0.0360 |
| FBP1 | 0.20 | 0.60 | 2.98 | down | 0.0303 |
| CYP4F3 | 0.08 | 0.24 | 2.97 | down | 0.0152 |
| SPAG4 | 0.72 | 2.15 | 2.97 | down | 0.0137 |
| AIFM3 | 0.22 | 0.67 | 2.96 | down | 0.0278 |
| SERPINE1 | 0.09 | 0.26 | 2.95 | down | 0.0354 |
| ST3GAL6 | 0.35 | 1.02 | 2.94 | down | 0.0238 |
| PDSS1 | 5.22 | 15.26 | 2.93 | down | 0.0313 |
| ABCC3 | 0.08 | 0.24 | 2.92 | down | 0.0006 |
| NEFM | 0.51 | 1.47 | 2.90 | down | 0.0210 |
| TMEM246 | 0.35 | 1.00 | 2.84 | down | 0.0489 |
| PCDHGC3 | 0.06 | 0.18 | 2.80 | down | 0.0483 |
| CXCR2 | 0.22 | 0.57 | 2.64 | down | 0.0131 |
| HSPA4L | 3.39 | 8.89 | 2.62 | down | 0.0292 |
| SPINK9 | 0.55 | 1.44 | 2.61 | down | 0.0231 |
| RBPMS2 | 0.45 | 1.17 | 2.59 | down | 0.0455 |
| GPNMB | 0.03 | 0.07 | 2.57 | down | 0.0466 |
| NOTCH3 | 0.04 | 0.09 | 2.50 | down | 0.0424 |
| CRADD | 0.92 | 2.28 | 2.49 | down | 0.0246 |
| AK8 | 1.33 | 3.26 | 2.46 | down | 0.0145 |
| LAMB3 | 3.08 | 7.54 | 2.45 | down | 0.0189 |
| G0S2 | 8.24 | 20.16 | 2.45 | down | 0.0133 |
| QPCT | 0.85 | 2.08 | 2.44 | down | 0.0230 |
| CLTCL1 | 0.45 | 1.09 | 2.42 | down | 0.0428 |
| C14orf132 | 0.21 | 0.51 | 2.41 | down | 0.0414 |
| MMEL1 | 0.51 | 1.22 | 2.40 | down | 0.0469 |
| LRRC4 | 0.10 | 0.23 | 2.40 | down | 0.0156 |
| TBC1D7 | 0.87 | 2.07 | 2.39 | down | 0.0129 |
| TDRD15 | 0.60 | 1.43 | 2.38 | down | 0.0135 |
| TNNT2 | 0.73 | 1.70 | 2.32 | down | 0.0382 |
| SCARF1 | 3.99 | 9.26 | 2.32 | down | 0.0204 |
| MSANTD3 | 1.63 | 3.79 | 2.32 | down | 0.0299 |
| F10 | 0.58 | 1.34 | 2.30 | down | 0.0498 |
| CCR9 | 0.43 | 0.99 | 2.29 | down | 0.0080 |
| KBTBD11 | 0.85 | 1.92 | 2.25 | down | 0.0340 |
| PINLYP | 0.37 | 0.83 | 2.24 | down | 0.0132 |
| OR1D5 | 0.50 | 1.10 | 2.20 | down | 0.0413 |
| SOWAHC | 1.30 | 2.81 | 2.16 | down | 0.0417 |
| ZSCAN5A | 0.87 | 1.86 | 2.13 | down | 0.0395 |
| ENC1 | 0.46 | 0.99 | 2.13 | down | 0.0103 |
| GOLGA8T | 0.77 | 1.64 | 2.12 | down | 0.0132 |
| GPR157 | 0.99 | 2.11 | 2.12 | down | 0.0246 |
| SLC4A11 | 0.50 | 1.05 | 2.11 | down | 0.0240 |
| MS4A4A | 0.10 | 0.20 | 2.11 | down | 0.0238 |
| HOXA9 | 0.43 | 0.91 | 2.11 | down | 0.0305 |
| CILP | 0.66 | 1.37 | 2.08 | down | 0.0018 |
| BMP1 | 0.71 | 1.47 | 2.07 | down | 0.0011 |
| ADC | 0.79 | 1.62 | 2.06 | down | 0.0380 |
| ANKRD66 | 1.15 | 2.37 | 2.05 | down | 0.0457 |
| FGFR1 | 0.46 | 0.94 | 2.03 | down | 0.0196 |
| STOX1 | 1.01 | 2.06 | 2.03 | down | 0.0423 |
| DAB2 | 0.26 | 0.52 | 2.01 | down | 0.0006 |
| ARHGEF18 | 0.42 | 0.78 | 1.88 | down | 0.0389 |
| **ATB/CON** | **ATB** | **CON** | **Ratio** | **Regulation** | ***P* value** |
| GPRC5A | 11.77 | 0.35 | 50.34 | up | 0.0099 |
| IFNG | 31.20 | 4.52 | 6.90 | up | 0.0231 |
| ENPP3 | 4.99 | 0.79 | 6.34 | up | 0.0497 |
| SLC6A9 | 6.89 | 1.22 | 5.64 | up | 0.0396 |
| IL26 | 3.63 | 0.95 | 5.09 | up | 0.0481 |
| FAM66A | 0.83 | 0.35 | 4.75 | up | 0.0265 |
| CXCL10 | 15.41 | 3.41 | 4.52 | up | 0.0134 |
| MYBPH | 5.47 | 1.66 | 4.38 | up | 0.0281 |
| CCL4L2 | 6.22 | 1.42 | 4.38 | up | 0.0203 |
| CXCL11 | 5.43 | 1.24 | 4.38 | up | 0.0343 |
| XAF1 | 9.05 | 2.08 | 4.35 | up | 0.0338 |
| DNAH11 | 1.88 | 0.34 | 4.16 | up | 0.0361 |
| EDN1 | 13.01 | 3.23 | 4.03 | up | 0.0498 |
| RASD2 | 3.85 | 0.97 | 3.98 | up | 0.0454 |
| ANLN | 0.74 | 0.19 | 3.98 | up | 0.0078 |
| BCAN | 2.39 | 0.60 | 3.95 | up | 0.0040 |
| TIMP3 | 0.68 | 0.17 | 3.88 | up | 0.0017 |
| KIRREL2 | 1.84 | 0.49 | 3.76 | up | 0.0337 |
| MYOF | 1.69 | 0.46 | 3.71 | up | 0.0201 |
| MTRNR2L1 | 0.53 | 0.14 | 3.69 | up | 0.0098 |
| MMP1 | 4.95 | 1.35 | 3.67 | up | 0.0195 |
| CFHR4 | 1.83 | 0.50 | 3.62 | up | 0.0026 |
| SNCAIP | 1.64 | 0.46 | 3.58 | up | 0.0354 |
| MEDAG | 4.30 | 1.20 | 3.57 | up | 0.0014 |
| LHFP | 0.73 | 0.21 | 3.53 | up | 0.0252 |
| MAOA | 1.66 | 0.47 | 3.53 | up | 0.0282 |
| CXCL5 | 6.19 | 1.77 | 3.49 | up | 0.0269 |
| RNF19B | 13.88 | 3.79 | 3.48 | up | 0.0236 |
| SOCS2 | 2.12 | 0.62 | 3.40 | up | 0.0363 |
| TMEM88 | 5.50 | 1.66 | 3.32 | up | 0.0043 |
| NCAPG | 0.85 | 0.26 | 3.21 | up | 0.0177 |
| PKMYT1 | 0.60 | 0.19 | 3.17 | up | 0.0249 |
| CDCP2 | 0.86 | 0.27 | 3.15 | up | 0.0137 |
| KCNT2 | 6.59 | 2.21 | 2.99 | up | 0.0040 |
| DUSP27 | 0.42 | 0.15 | 2.85 | up | 0.0480 |
| MYBL2 | 1.79 | 0.64 | 2.81 | up | 0.0393 |
| CRISPLD2 | 0.64 | 0.24 | 2.70 | up | 0.0055 |
| IL1RN | 9.15 | 3.54 | 2.59 | up | 0.0056 |
| IL5 | 1.58 | 0.61 | 2.58 | up | 0.0108 |
| ALOX5 | 1.92 | 0.75 | 2.56 | up | 0.0310 |
| SDC1 | 2.14 | 0.84 | 2.55 | up | 0.0164 |
| CLMP | 1.31 | 5.14 | 2.55 | up | 0.0061 |
| SRXN1 | 5.11 | 2.01 | 2.54 | up | 0.0015 |
| HTR1F | 0.87 | 0.37 | 2.37 | up | 0.0420 |
| HYDIN | 7.76 | 3.40 | 2.28 | up | 0.0116 |
| TLR7 | 5.31 | 2.35 | 2.25 | up | 0.0271 |
| GBP4 | 2.66 | 1.20 | 2.23 | up | 0.0256 |
| HSH2D | 6.86 | 3.13 | 2.19 | up | 0.0038 |
| STAT5A | 10.38 | 4.76 | 2.18 | up | 0.0015 |
| TRPV1 | 0.61 | 0.29 | 2.13 | up | 0.0049 |
| SLCO1A2 | 2.10 | 0.98 | 2.13 | up | 0.0184 |
| CPXM1 | 2.14 | 1.02 | 2.10 | up | 0.0463 |
| MLTK | 17.85 | 8.65 | 2.06 | up | 0.0402 |
| ASCL4 | 0.71 | 0.35 | 2.06 | up | 0.0132 |
| GPR64 | 0.18 | 1.08 | 7.79 | down | 0.0431 |
| P4HA3 | 0.36 | 2.74 | 7.62 | down | 0.0237 |
| ANKRD35 | 0.15 | 1.55 | 7.58 | down | 0.0009 |
| APCDD1L | 0.32 | 1.78 | 7.49 | down | 0.0061 |
| FAM187A | 0.24 | 1.23 | 6.80 | down | 0.0354 |
| VCAN | 0.08 | 0.45 | 5.94 | down | 0.0420 |
| GPR146 | 0.31 | 1.78 | 5.67 | down | 0.0173 |
| SLC12A5 | 0.21 | 1.04 | 5.01 | down | 0.0193 |
| WEE2 | 0.35 | 1.24 | 4.79 | down | 0.0062 |
| IER5L | 0.18 | 0.87 | 4.79 | down | 0.0223 |
| NMUR1 | 0.45 | 1.75 | 3.90 | down | 0.0247 |
| STAB1 | 0.83 | 3.12 | 3.78 | down | 0.0322 |
| PTPRB | 0.12 | 0.44 | 3.74 | down | 0.0465 |
| SMKR1 | 1.45 | 5.27 | 3.63 | down | 0.0156 |
| FAM131B | 0.93 | 3.23 | 3.48 | down | 0.0289 |
| POU3F1 | 0.45 | 1.56 | 3.44 | down | 0.0062 |
| TGFBI | 4.92 | 16.63 | 3.38 | down | 0.0326 |
| EEPD1 | 0.95 | 3.14 | 3.32 | down | 0.0096 |
| NPTX1 | 0.52 | 1.69 | 3.22 | down | 0.0200 |
| EPHA1 | 2.77 | 8.84 | 3.19 | down | 0.0353 |
| ANGPT4 | 0.01 | 0.04 | 3.05 | down | 0.0143 |
| GJA3 | 0.53 | 1.57 | 2.98 | down | 0.0269 |
| PMP22 | 2.06 | 6.00 | 2.92 | down | 0.0098 |
| DTHD1 | 4.27 | 11.88 | 2.78 | down | 0.0456 |
| PDK4 | 0.35 | 0.98 | 2.78 | down | 0.0274 |
| SPIRE2 | 0.52 | 1.42 | 2.74 | down | 0.0070 |
| TPSG1 | 0.03 | 0.08 | 2.67 | down | 0.0361 |
| TSPAN32 | 2.57 | 6.73 | 2.62 | down | 0.0027 |
| MMP23B | 2.10 | 5.47 | 2.60 | down | 0.0044 |
| C21orf90 | 0.11 | 0.28 | 2.52 | down | 0.0101 |
| SLC37A2 | 2.33 | 5.80 | 2.49 | down | 0.0156 |
| EFNA5 | 0.24 | 0.59 | 2.47 | down | 0.0426 |
| AGAP1 | 0.54 | 1.29 | 2.40 | down | 0.0045 |
| CPNE7 | 1.07 | 2.51 | 2.35 | down | 0.0388 |
| PCDHGC4 | 0.01 | 0.03 | 2.33 | down | 0.0117 |
| RGS18 | 9.63 | 22.35 | 2.32 | down | 0.0066 |
| SPTBN5 | 2.05 | 4.71 | 2.29 | down | 0.0235 |
| FOXD2 | 0.48 | 1.06 | 2.24 | down | 0.0345 |
| KCNE2 | 0.05 | 0.12 | 2.23 | down | 0.0042 |
| DAPK2 | 3.59 | 7.93 | 2.20 | down | 0.0304 |
| KLHL32 | 0.53 | 1.16 | 2.19 | down | 0.0071 |
| NPIP | 3.42 | 7.47 | 2.19 | down | 0.0025 |
| ATP8B3 | 1.07 | 2.29 | 2.15 | down | 0.0211 |
| FN3K | 0.56 | 1.18 | 2.12 | down | 0.0043 |
| **LTBI/CON** | **LTBI** | **CON** | **Ratio** | **Regulation** | ***P* value** |
| MOV10L1 | 2.25 | 0.19 | 11.97 | up | 0.0459 |
| TSACC | 4.07 | 0.55 | 7.34 | up | 0.0480 |
| NUP210L | 0.82 | 0.13 | 6.22 | up | 0.0271 |
| GALT | 2.57 | 0.43 | 6.01 | up | 0.0059 |
| ZNF843 | 1.68 | 0.30 | 5.65 | up | 0.0356 |
| GAB4 | 1.15 | 0.22 | 5.31 | up | 0.0257 |
| GGT5 | 80.22 | 16.42 | 4.88 | up | 0.0123 |
| KIAA1045 | 0.95 | 0.20 | 4.76 | up | 0.0176 |
| ZBED2 | 4.72 | 1.01 | 4.68 | up | 0.0354 |
| DDR2 | 2.75 | 0.64 | 4.28 | up | 0.0200 |
| HCAR2 | 16.24 | 3.83 | 4.24 | up | 0.0461 |
| CCDC173 | 1.37 | 0.33 | 4.18 | up | 0.0424 |
| HSPA4L | 8.89 | 2.15 | 4.13 | up | 0.0225 |
| PDSS1 | 15.26 | 3.72 | 4.10 | up | 0.0278 |
| CD40 | 0.19 | 0.05 | 4.00 | up | 0.0375 |
| PIM1 | 11.68 | 2.94 | 3.97 | up | 0.0243 |
| IL15RA | 14.75 | 3.80 | 3.88 | up | 0.0353 |
| PLEK | 0.43 | 0.11 | 3.78 | up | 0.0143 |
| ANLN | 1.83 | 0.49 | 3.76 | up | 0.0269 |
| CLEC4D | 2.75 | 0.73 | 3.75 | up | 0.0456 |
| P2RY14 | 0.92 | 0.25 | 3.74 | up | 0.0274 |
| TFRC | 11.98 | 3.23 | 3.71 | up | 0.0361 |
| LINC00853 | 1.57 | 0.43 | 3.65 | up | 0.0193 |
| IL31RA | 0.38 | 0.11 | 3.62 | up | 0.0101 |
| TXN | 4.04 | 1.12 | 3.60 | up | 0.0156 |
| EBI3 | 1.72 | 0.48 | 3.59 | up | 0.0426 |
| SLAMF7 | 1.28 | 0.36 | 3.58 | up | 0.0388 |
| TNFRSF9 | 13.97 | 3.98 | 3.51 | up | 0.0117 |
| HTR1F | 1.96 | 0.56 | 3.49 | up | 0.0235 |
| P2RX7 | 0.68 | 0.20 | 3.47 | up | 0.0345 |
| DCUN1D3 | 3.12 | 0.91 | 3.44 | up | 0.0304 |
| C4A | 2.34 | 0.69 | 3.40 | up | 0.0211 |
| LITAF | 2.54 | 0.75 | 3.38 | up | 0.0172 |
| LDLRAD2 | 2.06 | 0.63 | 3.30 | up | 0.0459 |
| GBP7 | 0.43 | 0.13 | 3.28 | up | 0.0466 |
| TREM2 | 0.53 | 0.16 | 3.23 | up | 0.0225 |
| SOCS2 | 5.65 | 1.79 | 3.15 | up | 0.0459 |
| GBP4 | 0.48 | 0.16 | 3.07 | up | 0.0364 |
| SATL1 | 1.25 | 0.41 | 3.06 | up | 0.0150 |
| CHST1 | 7.42 | 2.45 | 3.03 | up | 0.0388 |
| SERPINB9 | 2.10 | 0.70 | 3.01 | up | 0.0130 |
| NES | 1.20 | 0.40 | 3.01 | up | 0.0247 |
| GCH1 | 7.13 | 2.40 | 2.97 | up | 0.0465 |
| CRADD | 1.14 | 0.39 | 2.94 | up | 0.0110 |
| IRF8 | 4.40 | 1.50 | 2.93 | up | 0.0481 |
| LMCD1 | 1.68 | 0.58 | 2.91 | up | 0.0477 |
| NBN | 0.46 | 0.16 | 2.84 | up | 0.0218 |
| RIPK2 | 1.20 | 0.43 | 2.81 | up | 0.0222 |
| IL24 | 3.10 | 1.11 | 2.79 | up | 0.0253 |
| TREML2 | 3.78 | 1.39 | 2.72 | up | 0.0478 |
| LIMK1 | 0.29 | 0.11 | 2.66 | up | 0.0164 |
| TGM2 | 11.51 | 4.46 | 2.58 | up | 0.0226 |
| GGT5 | 2.20 | 0.88 | 2.51 | up | 0.0186 |
| HTR1F | 4.71 | 1.91 | 2.47 | up | 0.0330 |
| PCGF2 | 0.19 | 0.08 | 2.45 | up | 0.0170 |
| PDSS1 | 6.09 | 2.54 | 2.40 | up | 0.0137 |
| CPM | 7.22 | 3.02 | 2.39 | up | 0.0232 |
| MATN2 | 1.14 | 0.48 | 2.39 | up | 0.0355 |
| TIFA | 1.19 | 0.50 | 2.37 | up | 0.0295 |
| LTA | 0.43 | 0.18 | 2.36 | up | 0.0174 |
| LRG1 | 1.35 | 0.57 | 2.35 | up | 0.0322 |
| TNFRSF10D | 7.26 | 3.09 | 2.35 | up | 0.0408 |
| ROBO1 | 2.45 | 1.04 | 2.35 | up | 0.0418 |
| MYBL2 | 1.49 | 0.64 | 2.34 | up | 0.0498 |
| RGS16 | 0.26 | 0.11 | 2.31 | up | 0.0241 |
| TGM2 | 5.04 | 2.21 | 2.28 | up | 0.0283 |
| PPARG | 3.14 | 1.38 | 2.28 | up | 0.0116 |
| C7orf57 | 1.69 | 0.74 | 2.27 | up | 0.0484 |
| FOXC1 | 1.16 | 0.51 | 2.25 | up | 0.0036 |
| SLC22A4 | 4.92 | 2.19 | 2.24 | up | 0.0149 |
| SLC9B1 | 1.99 | 0.90 | 2.22 | up | 0.0286 |
| BMP10 | 0.14 | 0.06 | 2.22 | up | 0.0212 |
| SYT5 | 3.31 | 1.51 | 2.19 | up | 0.0096 |
| RASSF8 | 0.89 | 0.41 | 2.18 | up | 0.0142 |
| SLC17A3 | 1.23 | 0.57 | 2.17 | up | 0.0446 |
| KIF4A | 0.20 | 0.09 | 2.16 | up | 0.0448 |
| MAATS1 | 0.53 | 0.25 | 2.10 | up | 0.0453 |
| PALD1 | 1.48 | 0.72 | 2.07 | up | 0.0358 |
| LGMN | 0.43 | 0.21 | 2.06 | up | 0.0143 |
| FAM81B | 0.34 | 0.17 | 2.04 | up | 0.0137 |
| BHLHE22 | 0.06 | 0.03 | 2.04 | up | 0.0121 |
| RNF24 | 14.86 | 7.32 | 2.03 | up | 0.0114 |
| FBLN2 | 1.06 | 0.53 | 2.02 | up | 0.0283 |
| PNMT | 0.03 | 1.12 | 38.42 | down | 0.0456 |
| EPHX3 | 0.06 | 0.73 | 12.66 | down | 0.0434 |
| SCGB1B2P | 0.19 | 2.23 | 12.00 | down | 0.0112 |
| OLFML2A | 0.15 | 1.76 | 11.72 | down | 0.0419 |
| KCNJ10 | 0.16 | 1.12 | 7.00 | down | 0.0301 |
| MDH1B | 0.10 | 0.68 | 6.76 | down | 0.0004 |
| WIPF3 | 0.57 | 3.53 | 6.24 | down | 0.0073 |
| VSIG2 | 0.14 | 0.72 | 5.19 | down | 0.0306 |
| ANKRD35 | 0.27 | 1.16 | 4.37 | down | 0.0369 |
| EEPD1 | 0.06 | 0.27 | 4.32 | down | 0.0224 |
| OLR1 | 0.01 | 0.03 | 3.99 | down | 0.0417 |
| TKTL1 | 0.01 | 0.05 | 3.94 | down | 0.0320 |
| KLHL32 | 0.30 | 1.16 | 3.88 | down | 0.0415 |
| SIAH3 | 0.14 | 0.54 | 3.86 | down | 0.0146 |
| SYT4 | 0.77 | 2.94 | 3.81 | down | 0.0004 |
| CD300E | 0.13 | 0.49 | 3.77 | down | 0.0172 |
| SMCR2 | 0.05 | 0.17 | 3.62 | down | 0.0466 |
| TRAF2 | 0.03 | 0.12 | 3.61 | down | 0.0225 |
| GAL3ST4 | 0.75 | 2.72 | 3.61 | down | 0.0459 |
| REPS2 | 0.46 | 1.64 | 3.59 | down | 0.0364 |
| APOE | 0.08 | 0.27 | 3.50 | down | 0.0045 |
| CPA3 | 0.80 | 2.77 | 3.48 | down | 0.0152 |
| CACNA1C | 0.72 | 2.50 | 3.47 | down | 0.0284 |
| TPST1 | 0.44 | 1.50 | 3.44 | down | 0.0392 |
| FAM150B | 0.11 | 0.35 | 3.33 | down | 0.0431 |
| JUP | 0.11 | 0.37 | 3.31 | down | 0.0405 |
| SLC25A2 | 0.02 | 0.05 | 3.28 | down | 0.0049 |
| ARHGAP6 | 0.18 | 0.57 | 3.18 | down | 0.0162 |
| VWF | 0.05 | 0.17 | 3.13 | down | 0.0301 |
| GPC2 | 1.93 | 6.03 | 3.13 | down | 0.0215 |
| CCDC40 | 0.71 | 2.23 | 3.12 | down | 0.0226 |
| CHST7 | 1.36 | 4.24 | 3.11 | down | 0.0143 |
| NPFFR1 | 0.04 | 0.13 | 3.05 | down | 0.0298 |
| DRD3 | 0.69 | 2.08 | 3.03 | down | 0.0438 |
| MPP4 | 0.60 | 1.81 | 3.02 | down | 0.0375 |
| HCG23 | 0.03 | 0.10 | 2.97 | down | 0.0151 |
| NLRP6 | 0.47 | 1.35 | 2.89 | down | 0.0170 |
| GNG7 | 1.56 | 4.48 | 2.87 | down | 0.0218 |
| TBX1 | 0.04 | 0.10 | 2.82 | down | 0.0222 |
| TBXA2R | 0.63 | 1.75 | 2.79 | down | 0.0253 |
| GRIP1 | 0.13 | 0.36 | 2.79 | down | 0.0478 |
| FOXO6 | 0.06 | 0.18 | 2.76 | down | 0.0164 |
| C10orf131 | 1.35 | 3.68 | 2.72 | down | 0.0127 |
| CPLX1 | 0.51 | 1.37 | 2.71 | down | 0.0178 |
| BIRC7 | 0.21 | 0.55 | 2.69 | down | 0.0226 |
| EFNA1 | 0.28 | 0.76 | 2.67 | down | 0.0164 |
| RADIL | 0.47 | 1.25 | 2.64 | down | 0.0420 |
| GCSAML | 0.39 | 1.01 | 2.57 | down | 0.0116 |
| FOXJ1 | 0.15 | 0.38 | 2.54 | down | 0.0271 |
| HDC | 2.39 | 5.99 | 2.50 | down | 0.0256 |
| MTUS1 | 0.66 | 1.65 | 2.48 | down | 0.0184 |
| CTGF | 0.19 | 0.48 | 2.45 | down | 0.0463 |
| TAL1 | 0.17 | 0.41 | 2.42 | down | 0.0402 |
| HOXA4 | 0.15 | 0.37 | 2.41 | down | 0.0132 |
| ENO1-AS1 | 0.11 | 0.26 | 2.38 | down | 0.0272 |
| LINGO3 | 3.33 | 7.86 | 2.36 | down | 0.0424 |
| RYR1 | 0.39 | 0.92 | 2.33 | down | 0.0265 |
| SLC5A5 | 0.18 | 0.42 | 2.30 | down | 0.0152 |
| SYNM | 2.57 | 5.88 | 2.29 | down | 0.0144 |
| TAP2 | 2.52 | 5.73 | 2.27 | down | 0.0498 |
| BAIAP3 | 0.75 | 1.71 | 2.27 | down | 0.0308 |
| ADSSL1 | 0.46 | 1.04 | 2.25 | down | 0.0110 |
| FN3K | 0.53 | 1.18 | 2.25 | down | 0.0326 |
| HIST1H2BD | 0.34 | 0.75 | 2.23 | down | 0.0093 |
| FBLN7 | 3.98 | 8.84 | 2.22 | down | 0.0319 |
| PM20D1 | 0.25 | 0.55 | 2.20 | down | 0.0247 |
| SUSD4 | 0.16 | 0.34 | 2.15 | down | 0.0322 |
| EFHB | 0.08 | 0.16 | 2.14 | down | 0.0465 |
| FGF9 | 0.37 | 0.78 | 2.11 | down | 0.0156 |
| PRRT1 | 0.73 | 1.52 | 2.09 | down | 0.0289 |
| PCDHGC5 | 0.55 | 1.14 | 2.08 | down | 0.0285 |

**Supplementary Table 4.** GO analysis of different groups.

| **Groups** | **Name** | **-lgP** |
| --- | --- | --- |
| ATB to CON increase | immune system process | 4.0000 |
|  | response to stimulus | 4.0000 |
|  | locomotion | 2.1427 |
|  | multi-organism process | 1.9666 |
|  | multicellular organismal process | 1.5186 |
|  | developmental process | 1.4034 |
|  | regulation of biological process | 1.3152 |
| ATB to CON decrease | apical part of cell | 3.1549 |
|  | enzyme activator activity | 2.4202 |
|  | pattern binding | 2.1739 |
|  | extracellular space | 1.8996 |
|  | extracellular region part | 1.6234 |
|  | cell recognition | 1.5214 |
|  | nitrogen compound metabolic process | 1.4056 |
|  | carbohydrate binding | 1.3726 |
|  | transferase activity | 1.3107 |
| LTBI to CON increase | immune system process | 4.0000 |
|  | molecular transducer activity | 3.5229 |
|  | death | 3.5229 |
|  | positive regulation of biological process | 2.1612 |
|  | cell killing | 2.1367 |
|  | response to stimulus | 1.7986 |
| LTBI to CON decrease | synapse | 2.2218 |
|  | synapse part | 1.4698 |
|  | extracellular region part | 1.3716 |
|  | anatomical structure formation | 1.3270 |
|  | locomotion | 1.3080 |
| ATB to LTBI increase | extracellular region | 2.6198 |
|  | extracellular region part | 1.6696 |
|  | multi-organism process | 1.5817 |
|  | multicellular organismal process | 1.5003 |
|  | synapse | 1.3429 |
|  | cell killing | 1.3063 |
|  | immune system process | 1.3028 |
| ATB to LTBI decrease | extracellular region | 2.6778 |
|  | extracellular region part | 2.0757 |
|  | biological adhesion | 1.7878 |
|  | enzyme regulator activity | 1.3947 |
|  | structural molecule activity | 1.3072 |

**Supplementary Table 5.** Validation results of selected differentially expressed genes in pair-wise comparisons in test set from Biomarker Identification Cohort by qRT-PCR.

| **GeneSymbol** | **Mean FC value** | | **Ratio** | **Mann-Whitney U** |
| --- | --- | --- | --- | --- |
| **ATB/LTBI** | **ATB** | **LTBI** | **ATB/LTBI** | ***P* value** |
| CXCL9 | 2.2 | 5.89 | 0.37 | 0.084 |
| IFNG | 6.63 | 2.07 | 3.2 | **0.0341** |
| CXCL10 | 4.43 | 1.36 | 3.26 | 0.6187 |
| CXCL11 | 0.91 | 2.37 | 0.38 | 0.2081 |
| OAS1 | 1.03 | 2.59 | 0.4 | 0.5007 |
| IFI44 | 1.82 | 1.22 | 1.49 | 0.2787 |
| CCSER1 | 0.67 | 1.11 | 0.6 | 0.0651 |
| PGM5 | 3.71 | 1.03 | 3.6 | **0.0213** |
| SCGB1B2P | 0.72 | 0.98 | 0.73 | 0.4293 |
| EBF3 | 3.1 | 0.71 | 4.37 | **0.0151** |
| SPOCK1 | 0.79 | 1.4 | 0.56 | 0.1514 |
| NAV2-AS1 | 0.62 | 1.69 | 0.37 | 0.1688 |
| TNFRSF10C | 0.15 | 1.49 | 0.1 | **<0.0001** |
| LRRC36 | 0.82 | 3.25 | 0.25 | 0.23 |
| LUM | 0.74 | 2.68 | 0.28 | 0.0651 |
| A2ML1 | 1.82 | 0.52 | 3.5 | **0.0434** |
| **ATB/CON** | **ATB** | **CON** | **ATB/CON** | ***P* value** |
| IFNG | 2.07 | 0.71 | 2.91 | **0.0115** |
| CXCL10 | 4.43 | 0.55 | 7.99 | **0.0185** |
| CXCL11 | 0.91 | 0.69 | 1.32 | 0.4173 |
| OAS1 | 1.03 | 0.63 | 1.62 | 0.2698 |
| TNFRSF10C | 0.15 | 0.47 | 0.33 | **0.042** |
| ENPP3 | 2.54 | 0.53 | 4.75 | **0.0321** |
| SLC6A9 | 1.42 | 0.99 | 1.43 | 0.6448 |
| MYBPH | 4.97 | 0.78 | 6.39 | **0.0124** |
| CCL4L2 | 2.1 | 1.2 | 1.74 | 0.6887 |
| XAF1 | 1.81 | 0.71 | 2.56 | 0.2563 |
| EDN1 | 0.67 | 0.23 | 2.95 | 0.0693 |
| IL26 | 0.73 | 0.22 | 3.38 | **0.0403** |
| IER5L | 0.44 | 0.76 | 0.58 | 0.5932 |
| GPR146 | 0.26 | 1.25 | 0.21 | **0.0321** |
| VCAN | 0.32 | 0.87 | 0.37 | **0.0476** |
| GPRC5A | 4.61 | 1.05 | 4.39 | **0.0436** |
| GPR64 | 0.81 | 2.45 | 0.33 | **0.0414** |
| A2ML1 | 1.82 | 0.58 | 3.13 | **0.0488** |
| EBF3 | 3.1 | 0.64 | 4.84 | **0.0231** |
| **LTBI/CON** | **LTBI** | **CON** | **LTBI/CON** | ***P* value** |
| CD1A | 3.21 | 0.85 | 3.78 | **0.0325** |
| HBEGF | 10.93 | 1.74 | 6.3 | **0.0021** |
| ZBED2 | 3.8 | 1.15 | 3.32 | **0.0263** |
| DDR2 | 1.69 | 0.84 | 2.01 | 0.2541 |
| HCAR2 | 5.44 | 1.39 | 3.92 | **0.0302** |
| HSPA4L | 3.23 | 1.8 | 1.8 | 0.2749 |
| PDSS1 | 6.73 | 2.35 | 2.86 | **0.0412** |
| EEPD1 | 0.47 | 1.35 | 0.35 | 0.1621 |
| KCNJ10 | 0.21 | 0.72 | 0.3 | **0.0485** |
| EPHX3 | 0.87 | 1 | 0.87 | 0.3541 |
| A2ML1 | 1.82 | 0.58 | 3.13 | 0.0688 |
| IFNG | 6.63 | 0.71 | 9.32 | **0.0051** |
| CXCL10 | 4.36 | 0.55 | 7.87 | **0.0134** |
| CXCL11 | 2.34 | 0.69 | 3.37 | 0.0932 |
| OAS1 | 2.59 | 0.63 | 4.08 | 0.1457 |
| TNFRSF10C | 1.49 | 0.47 | 3.2 | **0.0355** |

**Supplementary Table 6.** The sequence (5'-3') of primers of qRT-PCR.

| **Gene symbol** | **Forward primer** | **Reverse primer** | **Product length** |
| --- | --- | --- | --- |
| GAPDH | CTCTCTGCTCCTCCTGTTCGAC | TGAGCGATGTGGCTCGGCT | 69 |
| CXCL9 | ACACCCCACAGAAGTGCTTT | GAGTGTTCACCCATGTGGTACT | 149 |
| IFNG | TGTCGCCAGCAGCTAAAACA | GCAGGCAGGACAACCATTAC | 90 |
| CXCL10 | ACCAAATCAGCTGCTACTACTCC | GCAGGGTCAGAACATCCACTA | 135 |
| CXCL11 | TGCCCAAAGGAGTCCAACAA | TGCAACAAGTAAGAACGTGAAA | 107 |
| OAS1 | CATCCGCCTAGTCAAGCACT | TCCAAGACCGTCCGAAATCC | 156 |
| IFI44 | TGGGAGCTGGACCCTGTAA | TCCTCCCTTAGATTCCCTATTTGC | 119 |
| CCSER1 | CCCGACAGCATTCGACCTT | CATCCTGCTGGCCTAGAGTTT | 141 |
| PGM5 | GGAAGCAGAGTGTGGAGGAAA | AGGATTTGTCTGTGACCAGGG | 147 |
| SCGB1B2P | GGACACCTACATGGACACTG | TCGGCACACTGGTTGTTGAT | 157 |
| EBF3 | CGCTGATGAAAAATGCCTCC | AGTCTGATGGCTCATACCTCCT | 132 |
| SPOCK1 | GCAAAAGAAGGGGAACGTGG | TGCACTTGGATGTGTAGGAGT | 131 |
| NAV2-AS1 | AGTGAGCAGTTTCGGGTCAA | CACCAGTGAAGGTGTCTCTGT | 136 |
| TNFRSF10C | TTCCTGCACCATGACCAGAG | TACTGACTTGGACTTCCCCAC | 122 |
| LRRC36 | CTGCAACTCAGGGCAATGGT | GGATCGGACTGTGGACTGAC | 155 |
| LUM | GTGCATTTACTCTCTTCCTGGC | TGGGTAGCTTTCAGGGCAGT | 134 |
| A2ML1 | ATTCGAGCCAGCGTTCCATT | GTATTTTCAGGCCCACGTCC | 90 |
| ENPP3 | GCTTGGACTCAGGAAACTGGA | TCACCTCGGTCTTTACATGCC | 114 |
| SLC6A9 | GATCAGCCCCATGTTCAAAGG | GTTGGAGGCGTCCAGTACAC | 193 |
| MYBPH | ATCTGGACACATGATGGCTGT | GAGTCAGCACGTTGGGCTT | 104 |
| CCL4L2 | AGAAAACCTCTTTGCCACCA | GCAGACTTGCTTGCCTCTTT | 121 |
| XAF1 | GCTCCACGAGTCCTACTGTG | GTTCACTGCGACAGACATCTC | 107 |
| EDN1 | AGAGTGTGTCTACTTCTGCCA | CTTCCAAGTCCATACGGAACAA | 78 |
| IL26 | GCTGTTAGTCACTCTGTCTCTTG | GGACAATGTTCCCCTTGGGTA | 85 |
| IER5L | GCATACCCTACCCCTTCGGA | CCTGGTGCAAGTAGCCGTT | 109 |
| GPR146 | GGACGCCACACTATCTGATCC | AGTGCAGTAGCCCCAGGTAG | 90 |
| VCAN | GCAAGTGATGCGGGTCTTTAC | TTGCCGCCCTGTAGTGAAAC | 109 |
| GPRC5A | ATGGCTACAACAGTCCCTGAT | CCACCGTTTCTAGGACGATGC | 103 |
| GPR64 | ACATGGATGGGCCTAGAAGC | CCCCAACCGACAATGCAGAA | 104 |
| CD1A | CACCTGGATCGCATCCTTTTAC | CATTGCTGAAGTTTCCCCTGG | 146 |
| HBEGF | ATCGTGGGGCTTCTCATGTTT | TTAGTCATGCCCAACTTCACTTT | 86 |
| ZBED2 | TGCACTGTGGAAGCATCTGAA | CCCCAGTTACCCTCAATGCC | 132 |
| DDR2 | AGGAGGTCATGGCATCGAGTT | GGTTCCGCCAAGAGATCCA | 83 |
| HCAR2 | ATGTTGGCTATGAACCGCCAG | GCTGCTGTCCGATTGGAGA | 119 |
| HSPA4L | TGTACCCCGGCCTGTATATCA | ACGTTCGTGACTATCTGGCTC | 80 |
| PDSS1 | AACATCTGCCTGTCCAAATGTAT | CGAGTTTGAAAGGATCGGTGTAT | 104 |
| EEPD1 | CAAGAACCCTCAAGGCTCGAA | CGCCCCAAGACCAGTTATCC | 137 |
| KCNJ10 | CCAAGGTGTATTACAGTCAGACC | CTGCGACCATCTTTTGTCAGG | 97 |
| EPHX3 | TGTGGACTTGCGAGGCTATG | ATGCACTTCGAGTAACCCAGG | 120 |

**Supplementary Table 7.** ROC analytic results of the differentially expressed gene in discriminating between ATB and LTBI or CON group in Biomarker Validation Cohort.

| **Gene** | **AUC 95%CI** | **Cut-off value** | **Sensitivity %** | **95% CI** | **Specificity %** | **95% CI** | **Likelihood ratio** |
| --- | --- | --- | --- | --- | --- | --- | --- |
| ATB vs LTBI |  |  |  |  |  |  |  |
| *TNFRSF10C* | 0.8669 (0.7971-0.9368) | < 0.7967 | 78.43 | 64.68% to 88.71% | 84.09 | 69.93% to 93.36% | 4.931 |
| *PGM5* | 0.6911 (0.5690-0.7933) | > 3.931 | 56.86 | 42.25% to 70.65% | 86.36 | 72.65% to 94.83% | 4.17 |
| *A2ML1* | 0.8059 (0.7163-0.8956) | > 2.749 | 74.51 | 60.37% to 85.67% | 79.55 | 64.70% to 90.20% | 3.643 |
| *IFN-gamma* | 0.6878 (0.5795-0.7962) | > 11.64 | 54.90 | 40.34% to 68.87% | 84.09 | 69.93% to 93.36% | 3.451 |
| *EBF3* | 0.7885 (0.6945-0.8826) | > 3.281 | 68.63 | 54.11% to 80.89% | 84.09 | 69.93% to 93.36% | 4.314 |
| ATB vs (LTBI+CON) |  |  |  |  |  |  |  |
| *TNFRSF10C* | 0.7331 (0.6412-0.8193) | < 0.4555 | 52.94 | 38.46% to 67.07% | 83.54 | 73.51% to 90.94% | 3.217 |
| *PGM5* | 0.7154 (0.6071-0.8237) | > 3.262 | 62.75 | 48.08% to 75.87% | 88.61 | 79.47% to 94.66% | 5.508 |
| *A2ML1* | 0.8445 (0.7726-0.9164) | > 2.84 | 74.51 | 60.37% to 85.67% | 83.54 | 73.51% to 90.94% | 4.528 |
| *IFN-gamma* | 0.8187(0.7466-0.8908) | > 7.495 | 76.47 | 62.51% to 87.21% | 73.42 | 62.28% to 82.73% | 2.877 |
| *EBF3* | 0.7857(0.6991-0.8724) | > 2.605 | 74.51 | 60.37% to 85.67% | 78.48 | 67.8% to 86.94% | 3.463 |

**Supplementary Table 8.** ROC analytic results of the differentially expressed gene in discriminating between ATB patients and subjects without ATB in Biomarker Application Cohort.

| **Gene** | **AUC 95%CI** | **Cutoff value** | **Sensitivity %** | **95% CI** | **Specificity %** | **95% CI** | **Likelihood ratio** |
| --- | --- | --- | --- | --- | --- | --- | --- |
| ATB vs NoATB |  |  |  |  |  |  |  |
| *TNFRSF10C* | 0.8251(0.7274-0.9228) | < 2.215 | 86.76 | 72.57% to 95.70% | 64.56 | 44.07% to 81.36% | 2.441 |
| *A2ML1* | 0.7843(0.6706-0.8981) | > 2.192 | 79.41 | 63.54% to 90.70% | 68.35 | 47.65% to 84.12% | 2.473 |
| *EBF3* | 0.8086(0.6978-0.9194) | > 1.615 | 82.35 | 66.47% to 92.46% | 82.28 | 63.11% to 93.94% | 4.595 |

**Supplementary Table 9.** Diagnostic performances of the 3-gene signature set in BCG vaccinated and BCG unvaccinated patients in Clinical Application Cohort.

| **3-gene set** | **BCG vaccinated (n=109)** | | **BCG unvaccinated (n=38)** | | ***P*-value** |
| --- | --- | --- | --- | --- | --- |
|  | **ATB (n=53)** | **NoATB (n=56)** | **ATB (n=15)** | **NoATB (n=23)** |  |
| Sensitivity %, (n/N) | 81.1 (43/53) |  | 86.7(13/15) |  | 0.72 |
| Specificity %, (n/N) |  | 91.1(51/56) |  | 95.7(22/23) | 0.67 |
| Accuracy %, (n/N) | 86.2(94/109) |  | 92.1(35/38) |  | 0.41 |

# Supplementary Figures.

**Supplementary Figure 1.** Flow chart of the study process and T-SPOT conversion and reversion of household contacts at 6-year follow-up.


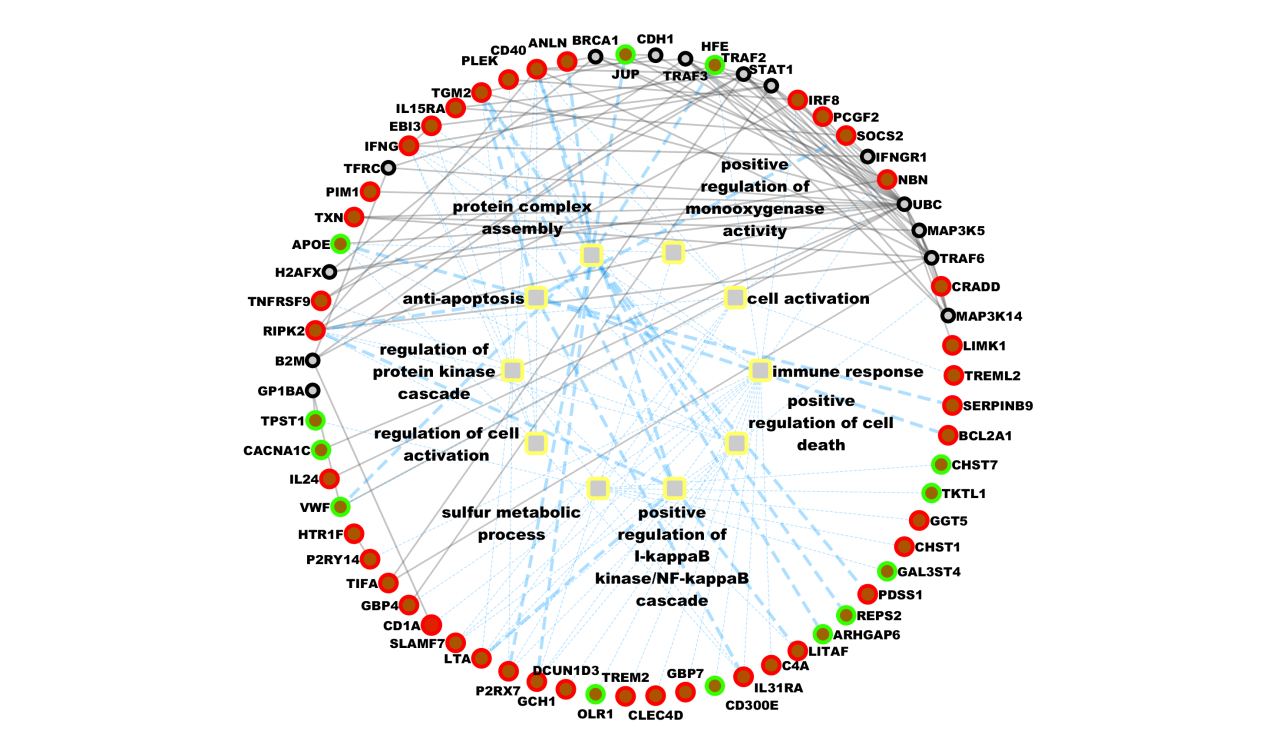


**Supplementary Figure 2.** Visualization of relationships of differential expressed genes between LTBI and CON group. Circle nodes represents genes, while Gray filled rectangle nodes with yellow border color indicate biological processes. For genes, borders of the nodes represent the type of the gene (up regulated in red, down regulated in green), the centers of the nodes indicate the gene expression changes, color intensity is proportional to the level of regulation. Genes that not quantified are shown in gray. Protein-protein interactions are depicted as grey solid line, dashed lines show the linkage of gene to related biological processes. Big gray circle indicates main module of biological processes.


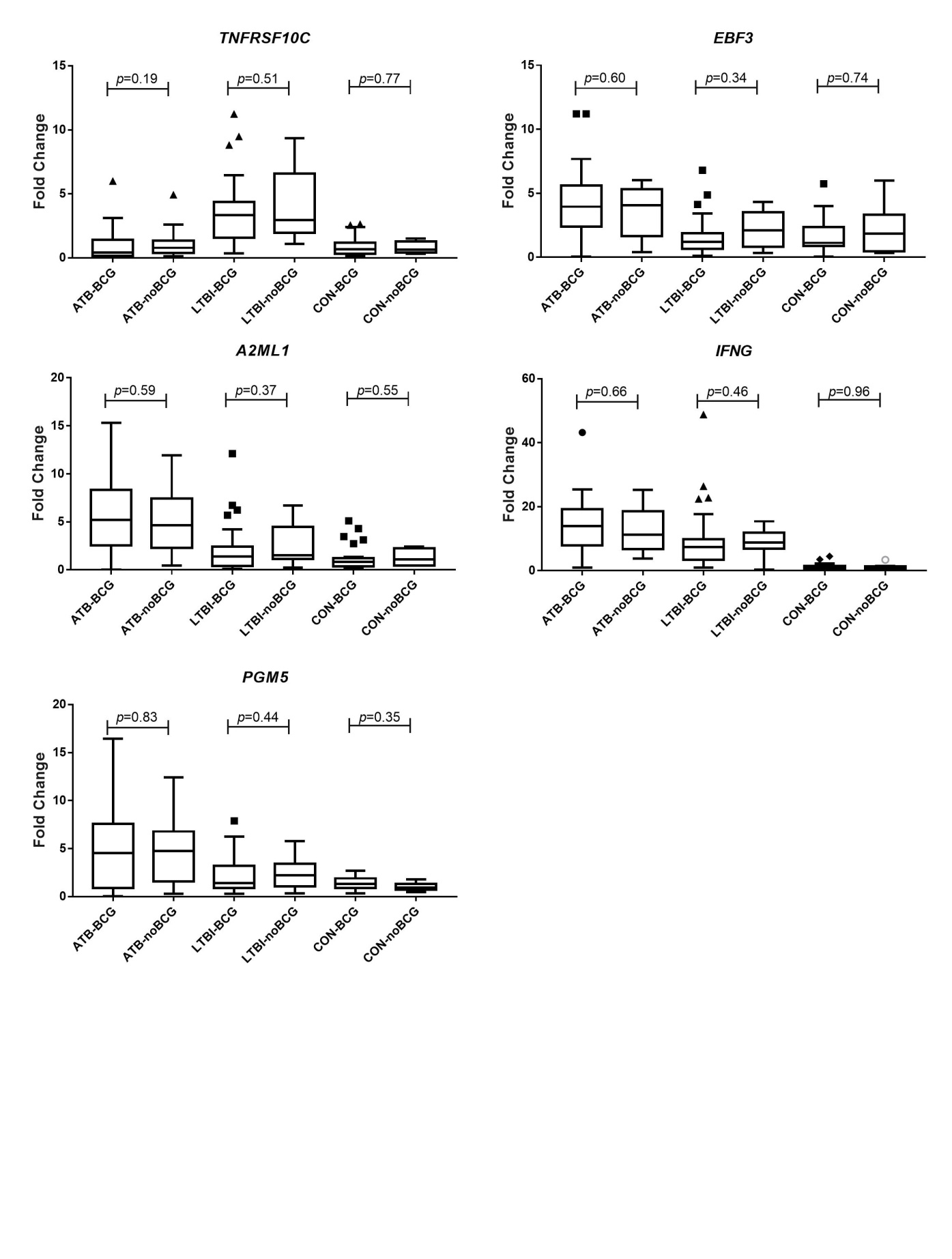


**Supplementary Figure 3.** PPD-stimulated gene expressions stratified by BCG vaccination in ATB, LTBI and CON group from the Biomarker Validation Cohort.

# References

1. Langmead B, Trapnell C, Pop M, Salzberg SL: **Ultrafast and memory-efficient alignment of short DNA sequences to the human genome**. *Genome biology* 2009, **10**(3):R25.

2. Trapnell C, Pachter L, Salzberg SL: **TopHat: discovering splice junctions with RNA-Seq**. *Bioinformatics (Oxford, England)* 2009, **25**(9):1105-1111.

3. Trapnell C, Roberts A, Goff L, Pertea G, Kim D, Kelley DR, Pimentel H, Salzberg SL, Rinn JL, Pachter L: **Differential gene and transcript expression analysis of RNA-seq experiments with TopHat and Cufflinks**. *Nature protocols* 2012, **7**(3):562-578.

4. Huang da W, Sherman BT, Lempicki RA: **Systematic and integrative analysis of large gene lists using DAVID bioinformatics resources**. *Nat Protoc* 2009, **4**(1):44-57.

5. Huang da W, Sherman BT, Lempicki RA: **Bioinformatics enrichment tools: paths toward the comprehensive functional analysis of large gene lists**. *Nucleic Acids Res* 2009, **37**(1):1-13.

6. Franceschini A, Szklarczyk D, Frankild S, Kuhn M, Simonovic M, Roth A, Lin J, Minguez P, Bork P, von Mering C *et al*: **STRING v9.1: protein-protein interaction networks, with increased coverage and integration**. *Nucleic Acids Res* 2013, **41**(Database issue):D808-815.

7. Cline MS, Smoot M, Cerami E, Kuchinsky A, Landys N, Workman C, Christmas R, Avila-Campilo I, Creech M, Gross B *et al*: **Integration of biological networks and gene expression data using Cytoscape**. *Nat Protoc* 2007, **2**(10):2366-2382.
